# Muscle-Inspired Anisotropic Aramid Nanofibers Aerogel Exhibiting High-Efficiency Thermoelectric Conversion and Precise Temperature Monitoring for Firefighting Clothing

**DOI:** 10.1007/s40820-025-01728-x

**Published:** 2025-04-14

**Authors:** Zhicai Yu, Yuhang Wan, Mi Zhou, Md Hasib Mia, Siqi Huo, Lele Huang, Jie Xu, Qing Jiang, Zhenrong Zheng, Xiaodong Hu, Hualing He

**Affiliations:** 1 State Key Laboratory of New Textile Materials and Advanced Processing, School of Textile Science and Engineering, Wuhan Textile University, Wuhan, 430200 People’s Republic of China; 2 School of Engineering, Centre for Future Materials, University of Southern Queensland, Springfield Central, 4300 Australia; 3 School of Textile Science and Engineering, Tiangong University, Tianjin, 300387 People’s Republic of China; 4 College of Material and Chemical Engineering, Hunan Institute of Engineering, Xiangtan, 411104 People’s Republic of China

**Keywords:** Human muscle inspired, Anisotropic thermoelectric aerogel, Temperature sensing, High-temperature warning, Firefighting clothing

## Abstract

Enhancing the firefighting protective clothing with exceptional thermal barrier and temperature sensing functions to ensure high fire safety for firefighters has long been anticipated, but it remains a major challenge. Herein, inspired by the human muscle, an anisotropic fire safety aerogel (ACMCA) with precise self-actuated temperature monitoring performance is developed by combining aramid nanofibers with eicosane/MXene to form an anisotropically oriented conductive network. By combining the two synergies of the negative temperature-dependent thermal conductive eicosane, which induces a high-temperature differential, and directionally ordered MXene that establishes a conductive network along the directional freezing direction. The resultant ACMCA exhibited remarkable thermoelectric properties, with S values reaching 46.78 μV K^−1^ and *κ* values as low as 0.048 W m^−1^ K^−1^ at room temperature. Moreover, the prepared anisotropic aerogel ACMCA exhibited electrical responsiveness to temperature variations, facilitating its application in intelligent temperature monitoring systems. The designed anisotropic aerogel ACMCA could be incorporated into the firefighting clothing as a thermal barrier layer, demonstrating a wide temperature sensing range (50–400 °C) and a rapid response time for early high-temperature alerts (~ 1.43 s). This work provides novel insights into the design and application of temperature-sensitive anisotropic aramid nanofibers aerogel in firefighting clothing.

**Supplementary Information:**

The online version contains supplementary material available at 10.1007/s40820-025-01728-x.

## Highlights

- Muscle-inspired anisotropic aramid nanofibers thermoelectric aerogel was prepared via directional freeze-drying strategy.
- Resultant anisotropic aerogel exhibited high-efficiency thermoelectric conversion with Seebeck coefficient of 46.78 μV K^−1^ and low thermal conductivity of 0.048 W m^−1^ K^−1^.
- Thermoelectric aerogel-based multistage wireless high-temperature alarm system achieves a wide temperature sensing range (50–400 °C) and sensitivity response time (trigger time ~ 1.43 s) for firefighting clothing.

**Supplementary Information:**

The online version contains supplementary material available at 10.1007/s40820-025-01728-x.

## Graphical abstract

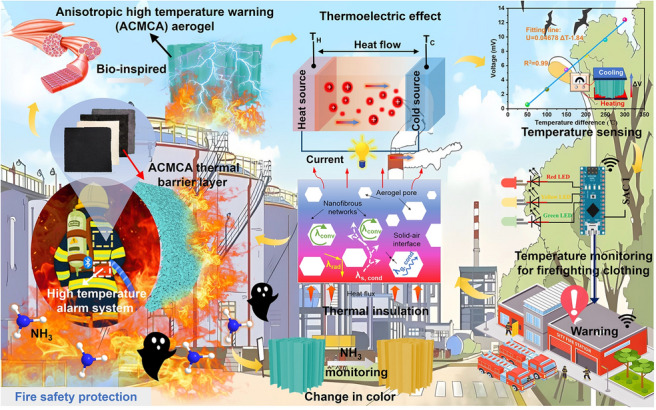

## Introduction

Firefighting clothing is essential equipment for protecting the safety of firefighters during fire rescue operations under extreme temperature conditions. The thermal protective performance of firefighting clothing primarily relies on thermal barrier layer in the multilayered fabric system, which can effectively protect firefighters from environmental thermal hazards during high heat exposure. Aerogels exhibit exceptional low density and thermal insulation performance, positioning them as the most promising candidates for high-efficiency thermal protection in firefighting clothing [1–6]. Among them, aramid nanofibers (ANFs)-based aerogel is the preferred choice for thermal barriers in firefighting clothing due to its superior thermostability and pore structure at the nano- and microscales, which significantly suppresses thermal conduction and convection within the aerogel skeleton under high radiant heat exposure [7–10]. Unfortunately, the ANFs-based insulation layer in firefighting clothing may still undergo thermal decomposition and cracking when subjected to prolonged exposure to extreme temperatures (≥ 400 °C), thereby endangering the safety of firefighters [11–13]. Integrating the intelligent temperature sensing function into ANFs aerogel will offer a novel solution that enables real-time monitoring and eliminates any pyrolytic damage in firefighting clothing [14]. This proactive strategy can effectively ensure personal safety by providing early warnings for firefighters before the thermal decomposition of the insulation layer in extreme fire conditions, prolonging the lifespan of firefighting clothing [15, 16].

Recent advances in temperature-responsive materials have generated significant interest in the development of temperature-sensitive aerogels for firefighting clothing. To date, the traditional high-temperature warning aerogel, which is based on thermal resistance materials, suffers from a critical flaw: its electrical signal transmission is heavily dependent on an external power source [17–19]. Therefore, this type of aerogel not only enhances the complexity of the temperature sensing system but also poses a risk of power supply failure due to long-term exposure to high-temperature conditions. By contrast, thermoelectric aerogels are considered effective temperature sensing thermal barrier layers in firefighting clothing, capable of directly converting heat into electrical voltage upon exposure to high temperatures, with the voltage intensity directly related to changes in temperature differences [20–23]. Therefore, ANFs-based thermoelectric aerogels achieve the self-driven and intermittent temperature sensing performance without relying on an external power supply, drawing heightened interest in many emerging fields of temperature-sensitive materials [24–28]. The key index for evaluating the energy conversion efficiency of ANFs-based thermoelectric aerogel is the dimensionless thermoelectric value ZT, which is determined by the Seebeck coefficient (*S*), electrical conductivity (*σ*), thermal conductivity (*κ*), absolute temperature (*T*), and power factor (PF*, S*^*2*^*σ*), with the expression ZT = *S*^*2*^*σT/κ* [29–32]. The ideal thermoelectric material should have high conductivity and Seebeck coefficient while maintaining low thermal conductivity. Therefore, how to regulate the balance among these parameters and improve the sensitivity and accuracy of the output electrical voltage signal in ANFs-based thermoelectric aerogel has become a key technical challenge to promote ultra-sensitive temperature detection performance in high-temperature warning applications.

Through the analysis of dimensionless thermoelectric value formula, we gain insight into a dual strategy to improve the thermoelectric performance of ANFs-based thermoelectric aerogel: one strategy involves maximizing the reduction of its thermal conductivity, thereby increasing its absolute temperature difference. Eicosane is a typical negative temperature coefficient thermal switching phase transition material that demonstrated considerable negative temperature dependence. Compared with traditional phase change materials, the molecular configuration of eicosane transitions from a relatively ordered state to a disordered liquid state as temperature rises [33, 34]. This transformation amplifies the scattering effect in the heat transfer process and effectively restricts heat transfer in ANFs-based thermoelectric aerogel, thereby maximizing thermal impedance and absolute temperature difference when one side is exposed to high temperature. As a result, a high thermoelectric value is achieved. Another strategy focuses on improving the electrical conductivity of ANFs-based thermoelectric aerogel to optimize its warning performance at high temperatures. Traditional thermoelectric aerogels exhibit an inherent isotropic porous structure, resulting in mechanical limitations and the shortest discontinuous carrier transport pathways, which restrict their efficacy in sensitive temperature sensing applications [2, 35]. In nature, muscle tissue from humans or animals meets the above proposed requirements of anisotropy and ordered structure, which can lead to high mechanical strength and directional molecular transport channels. This serves as an inspiration for the development of ANFs-based thermoelectric aerogels with exceptional electrical and mechanical properties oriented in a single direction [36–39]. To date, MXene, an emerging 2D transition metal carbide/carbonitride, has unique thermoelectric effects and excellent electrical conductivity, garnering significant interest from researchers for the fabrication of MXene-based temperature-sensitive aerogels [40–42]. Nevertheless, the random stacking of MXene nanosheets will limit the full utilization of their surface area and electrical conductivity properties. MXene nanosheets can be assembled into a uniform and 3D conductive structure network to overcome this fatal flaw by using the strong interaction between the –COOH group from functionalized multi-walled carbon nanotubes (MWCNTs-F) and the –OH group on the surface of MXene nanosheets, which eliminates the original defects [43, 44]. This innovation provides the MXene-based ANFs thermoelectric aerogel with excellent electrical conductivity, adjustable mechanical properties, and ductility.

Herein, inspired by the human muscle, an anisotropic and robust ANFs/eicosane (C20)/MXene/MWCNTs/Ag NWs thermoelectric aerogel (designated as ACMCA) was fabricated by directional freeze-drying. Benefiting from the anisotropic structured arrangement, the resulting ACMCA exhibited exceptional mechanical property (tensile strength of 2.52 MPa, compressive strength of 0.21 MPa, and compressive strain of 80%) and direction-dependent electrical conductivity (perpendicular direction 0.625 S m^−1^). By combining the dual synergies of the negative temperature-dependent thermal conductivity of C20, which induces a significant temperature difference, and the directional highly ordered conductive network of MXene formed along the freezing direction, the ACMCA exhibited exceptional thermoelectric properties, with S values of up to 46.78 μV K^−1^ and κ values as low as 0.048 W m^−1^ K^−1^ at room temperature. Meanwhile, on the basis of the thermoelectric effect, the ACMCA aerogel could convert the temperature stimuli into the electrical voltage signals, exhibiting a wide temperature range (50–400 °C) and precise temperature sensing performance (*R*^*2*^ = 0.99). Moreover, the as-prepared anisotropic ACMCA as thermal barrier layer was integrated into firefighting clothing with and early high-temperature warning system, exhibiting rapid and repeatable high-temperature warning properties with a response time of ~ 1.43 s. This work presented a facile anisotropic design of high-performance robust thermal insulation ANF-based aerogels, with heat insulation and self-actuated temperature sensing functions for application in firefighting clothing, thereby offering intelligent safety protection for firefighters under extreme temperatures.

## Experimental Section

### Materials

Kevlar fiber was supplied by Shanghai Mingxi Industrial Co., Ltd. Titanium aluminum carbide (Ti_3_AlC_2_, 200 mesh, ≥ 98.0%) and lithium fluoride (LiF) powder were supplied by Shanghai McLean Biochemical Co., Ltd. Silver nitrate (AgNO_3_), ethylene glycol, potassium hydroxide (KOH), eicosane (C20), hydrochloric acid (HCl, 37%), ethanol, dimethyl sulfoxide (DMSO), sodium chloride (NaCl), and tert-butanol were purchased from Sinopharm Chemical Reagent Co., Ltd., China. Carbon nanotubes (MWCNTs, purity > 95 wt%, diameter = 20–30 nm, length = 10–30 μm, multi-wall) were purchased from Jiangsu XFNANO Material Technology Co., Ltd. Nitric acid (HNO_3_, 65%) and sulfuric acid (H_2_SO_4_, 98%) were supplied by Shanghai Lingfeng Chemical Reagent Co., Ltd. Poly (vinylpyrrolidone)(Mw = 1,300,000) was purchased from Aladdin Co., Ltd.

### Preparation of ANFs Dispersion

KOH (1.5 g) and Kevlar fiber (1.5 g) were added to a beaker containing DMSO (80 mL) and magnetically stirred for 7 days to ensure that the reaction was sufficient and evenly carried out. At room temperature, the Kevlar fiber gradually undergoes a significant deprotonation process, which is accompanied with a profound change in color, from the initial state to a deep red color.

### Preparation of ANFs-Based Composite Aerogel

The prepared silver nanowires (Ag NWs, 20 mg, 2.1 wt%) were uniformly mixed in deionized water (2 mL) and subjected to ultrasounded in an ice water bath for 30 min to obtain a uniform Ag NWs dispersion solution. The prepared multi-wall carbon nanotubes (20 mg, 2.1 wt%) and MXene (80 mg, 8.4 wt%) were uniformly mixed in a 1:4 mass ratio in the Ag NWs dispersion solution resulting in a well-dispersed mixed solution (0.2 mg mL^−1^). Subsequently, the above mixed solution was evenly mixed with ANFs solution (0.5625 g, 58.3 wt%) and eicosane (0.28 g, 29.1 wt%), and injected into the polytetrafluoroethylene mold. To create a directional pore structure, we positioned the mold on a copper plate, which was frozen in a liquid nitrogen atmosphere for 20 min. The frozen dispersion was soaked in a mixture of ethanol/acetic acid (7:3) for 48 h to form an aerogel precursor of a specific size (4.2 cm in diameter and 0.9 mm in thickness). To optimize its structure, we immersed the aerogel precursor in a mixture of water/tert-butanol for solvent exchange. Finally, the aerogel precursor was freeze-dried for 16 h to obtain ACMCA high thermoelectric aerogel. The same preparation method as ACMCA aerogel was used, but MWCNTs-F and Ag NWs were not added. We named this aerogel "ACM." For the ANFs-based aerogel with a non-anisotropic structure, it was freely frozen in the refrigerator (− 20 °C), and the rest of the preparation process used the same method as the ACMCA aerogel, which was named non-ACMCA. In addition, to realize the visual detection function for toxic gases (NH_3_), we soaked the ACMCA aerogel in a CH_3_NH_3_PBI_3_/DMSO mixture to ensure complete impregnation, followed by drying in a vacuum oven at 100 °C for 3 h to obtain the ACMCA-M gas sensor.

### Construction of Temperature Monitoring and Alarm System

The system was composed of an indicator control circuit and signal acquisition circuit, including voltage acquisition and processing, display circuit, indicator light, calibration button, communication module, voltage reference, power circuit, STM32 single-chip microcomputer, and other modules. The thermoelectric aerogel generated different voltage signals at different temperatures. The signal acquisition module outputted and converted the voltage signals and compared them with the preset voltage threshold (the corresponding output voltage threshold at 200 and 400 °C was 6.8 and 15.6 mV, respectively). When the temperature was below 200 °C, the green alarm light was activated. When the temperature was between 200 and 400 °C, the yellow alarm light was activated. When the temperature exceeded 400 °C, the red alarm light was activated. The wireless transmitter sent a control command to the terminal receiver, and the wireless terminal received the signal and the rescue plan could be proposed in time.

### Characterization

The morphology and microstructure of the samples were analyzed by X-ray energy spectroscopy (EDX) and scanning electron microscopy (SEM, E1856-C2B, USA). The molecular structure and chemical composition of the samples were studied by X-ray diffraction (XRD, D8 Advance, Germany) and Fourier transform infrared spectroscopy (FTIR, tensor27, Germany). X-ray photoelectron spectroscopy (XPS, Thermo Kalpha, USA) was used to characterize the composition and chemical valence states of each component. The mechanical properties of aerogels were evaluated by an electronic universal testing machine (Instron 3365, USA). According to the GB/T 1040 -GB/T 20671.7-2006 standard, the aerogel tensile test was carried out at a tensile speed of 25.0 mm min^−1^, with a sample size of the parallel section > 20 mm and a thickness greater than 2 mm. Discovery TGA 55 (TA Instrument, USA) was used to test the thermal stability of the sample in a nitrogen atmosphere, with a rising/cooling rate of 10 °C min^−1^ in the range of 30–100 °C. According to the standard of GB2406-80, the combustion behavior of materials was characterized by using an oxygen index instrument (TTech-GBT2406-1, TESTECH, China). The thermal conductivity of different samples was measured using the TPS2500 thermal constant analyzer from Hot Disk Inc. in accordance with the ISO 22007-2 test standard. A differential scanning calorimeter (DSC200F3, NETZSCH, Germany) was used to determine the thermal properties (heat storage capacity) of the sample at a temperature/cooling rate of 10 K min^−1^ in the range of 0–100 °C in a nitrogen atmosphere.

## Results and Discussion

### Fabrication of Anisotropic Thermoelectric ACMCA Aerogel

**Fig. 1:**
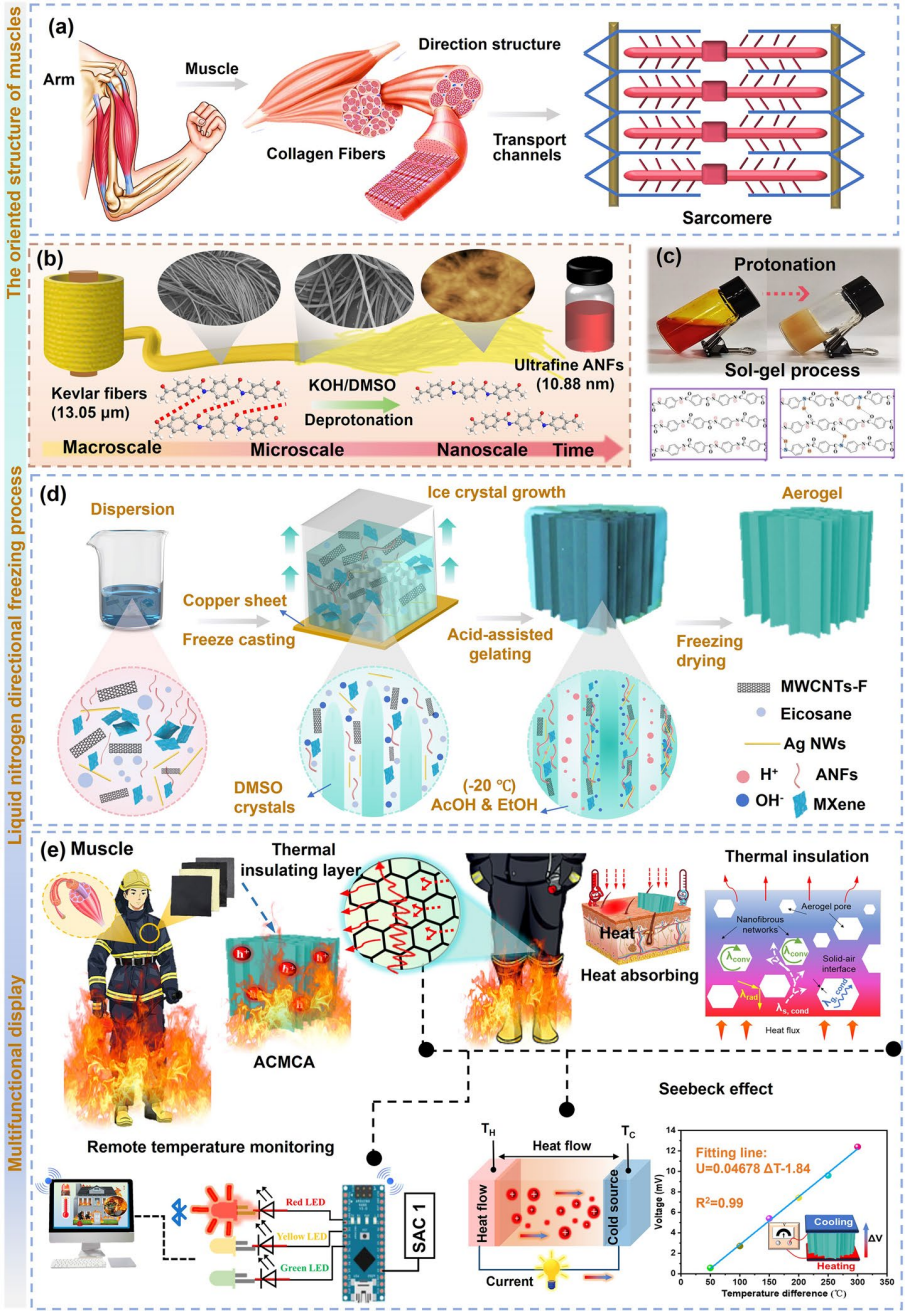
Concept description and preparation of ACMCA aerogel. **a** Schematic of the orientation structure of human muscle tissue. **b** Evolution process from macroscopic Kevlar fiber to ultra-fine ANFs. **c** Protonation process of ANFs dispersion solution. **d** Preparation of anisotropic aerogel ACMCA by directional freezing strategy. **e** Multifunctional demonstration of ACMCA aerogel with high thermal insulation and ultra-sensitive temperature sensing

This study involved the fabrication of muscle-inspired highly anisotropic and robust ANFs-based thermoelectric aerogel ACMCA using a sol–gel process and directional freezing using liquid nitrogen. Figure 1a shows a schematic of human muscle tissue characterized by a highly ordered structure (directional transport performance), facilitating the efficient transfer of energy, ions, and small molecules in a specific direction via internally arranged myofibrillar units. The following schematic illustrates our concept for designing these anisotropic thermoelectric aerogels. First, a proton donor-assisted deprotonation method was used to fabricate highly stable and homogeneous ANFs dispersion in a potassium hydroxide/dimethyl sulfoxide (KOH/DMSO) system, in which the macroscopic aramid fibers of poly-p-phenylene terephthalamide (PPTA) were split into individual ANFs with a large aspect ratio by weakening the hydrogen-bonding interactions among PPTA polymer chains (Fig. 1b). After a week of magnetic stirring, aramid fibers (average diameter of 13 μm and average length of 7 mm) yielded stable, deep red ANFs with a radial size of ~ 10.88 nm in a KOH/DMSO system, as evidenced by detailed observations from SEM and AFM images (Figs. S1 and S2). As shown in Fig. 1c, the obtained ANFs/DMSO dispersion underwent sol–gel transformation from a liquid state to a solid state after protonation process with the proton donor of H_2_O, thereby producing the ANFs aerogel precursor. In addition, the comparison of FTIR and XRD patterns in Fig. S3 showed that ANFs were highly consistent with the original PPTA fibers in terms of chemical structure and crystal morphology, suggesting that the deprotonation process did not destroy the structural integrity of the nanofibers.

Second, the efficient transport conductive thermoelectric MXene (Ti_3_C_2_T_x_) was prepared for the subsequent anisotropic aerogels that mimic ion and energy transport in specific directions of human muscles. In brief, the monolayer MXene was fabricated via selectively etching the Al atomic layer from the Ti_3_AlC_2_ precursor using a LiF/HCl etching solution (Fig. S4a–d). The surface of the etched MXene nanosheets was rich in –OH, –O, and –F groups, which could enhance the interfacial adhesion between MXene and ANFs in the aerogel through hydrogen-bonding interactions. Compared with Ti_3_AlC_2_, the XRD patterns of MXene showed that the strong crystallization (104) peak at 38.58° disappeared completely after the etching and delamination process, further implying that the successful etching of Al element from Ti_3_AlC_2_ (Fig. S4e). Furthermore, the (002) peak of MXene displayed a lower angle (5.02°) than that of Ti_3_AlC_2_ (9.56°), indicating an increased interlayer distance following the etching and delamination process. To overcome the agglomeration of the MXene/MWCNTs mixture in the present study, CNTs were treated with the H_2_SO_4_/HNO_3_ system as oxidants to obtain dispersed MWCNTs (MWCNTs-F) by introducing carboxyl and hydroxyl functional groups at the defect sites (Fig. S5). The FTIR spectrum of MWCNTs-F showed strong absorption peaks at 3467 and 1636 cm^−1^, corresponding to the stretching vibration peaks of the –OH and C=O groups, respectively, which strongly demonstrated the successful introduction of functional groups in the acid treatment process (Fig. S6).

As shown in Fig. 1d, functional doping of the aerogel was achieved by introducing functional materials into the ANFs dispersion to confer unique performance advantages. Inspired by the directional structure of human muscle tissue, we use the directional freeze-drying method to prepare anisotropic high thermal electrical gel. Different from the traditional isotropic structure, the anisotropic structure endows the aerogel with higher mechanical strength and continuous carrier transport path, which improves the performance of the aerogel in various applications. The aerogel precursor solution underwent directional freezing for 20 min at an extremely low temperature (− 198 °C) in a liquid nitrogen environment to achieve the directional development of the pore structure (Fig. S7). Given the unique physical properties of DMSO (melting point up to 18.4 °C), influenced by a vertical temperature gradient, DMSO typically forms dense and elongated columnar structures, a process that facilitates the rearrangement of ANFs into a dense and ordered skeleton. Subsequently, the frozen aerogel precursor blocks were submerged in a mixed coagulation bath of AcOH/EtOH (acetic acid and ethanol, volume ratio 7:3) at a low temperature (− 20 °C) for gelation to preserve the anisotropic microstructure of the aerogel precursor during solvent substitution. In this process, AcOH, as a proton donor, provided abundant hydrogen ions (H^+^), which enhanced the formation of hydrogen bonds between fibers, thereby achieving a high degree of regeneration and reinforcement of nanofibers. At the same time, the strategic addition of EtOH mitigated the excessive shrinkage of the skeleton induced by low surface tension, preserving the stability and integrity of the structure. After complete gelation, the ANFs-based composite hydrogel was sequentially washed with water and ethoxyacetic acid to remove the remaining AcOH and DMSO. In order to further optimize the performance, we used solvent exchange technology to replace the water part in the aerogel precursor with a tert-butanol/aqueous solution system, aiming to inhibit ice crystal formation during the subsequent freeze-drying process and preserve the microstructure of the aerogel (Fig. S8). Finally, highly cross-linked and directional aerogels were successfully prepared by freeze-drying. Figure S9 shows the digital image of the prepared ACMCA composite aerogel with high thermoelectric properties. The compliance (*r*_*b*_ = 2 mm) was satisfactory, and the lightweight density was 0.038 g cm^−3^. Figure 1e illustrates the innovative idea derived from the muscle structure found in nature, detailing the development of a multi-functional high thermoelectric (TE) aerogel. The figure indicates its broad application potential in wearable flexible sensors and firefighting clothing, showing the numerous possibilities presented by the integration of material science and bionic technology.

### Morphology and Structure of Anisotropic ACMCA Aerogel

**Fig. 2:**
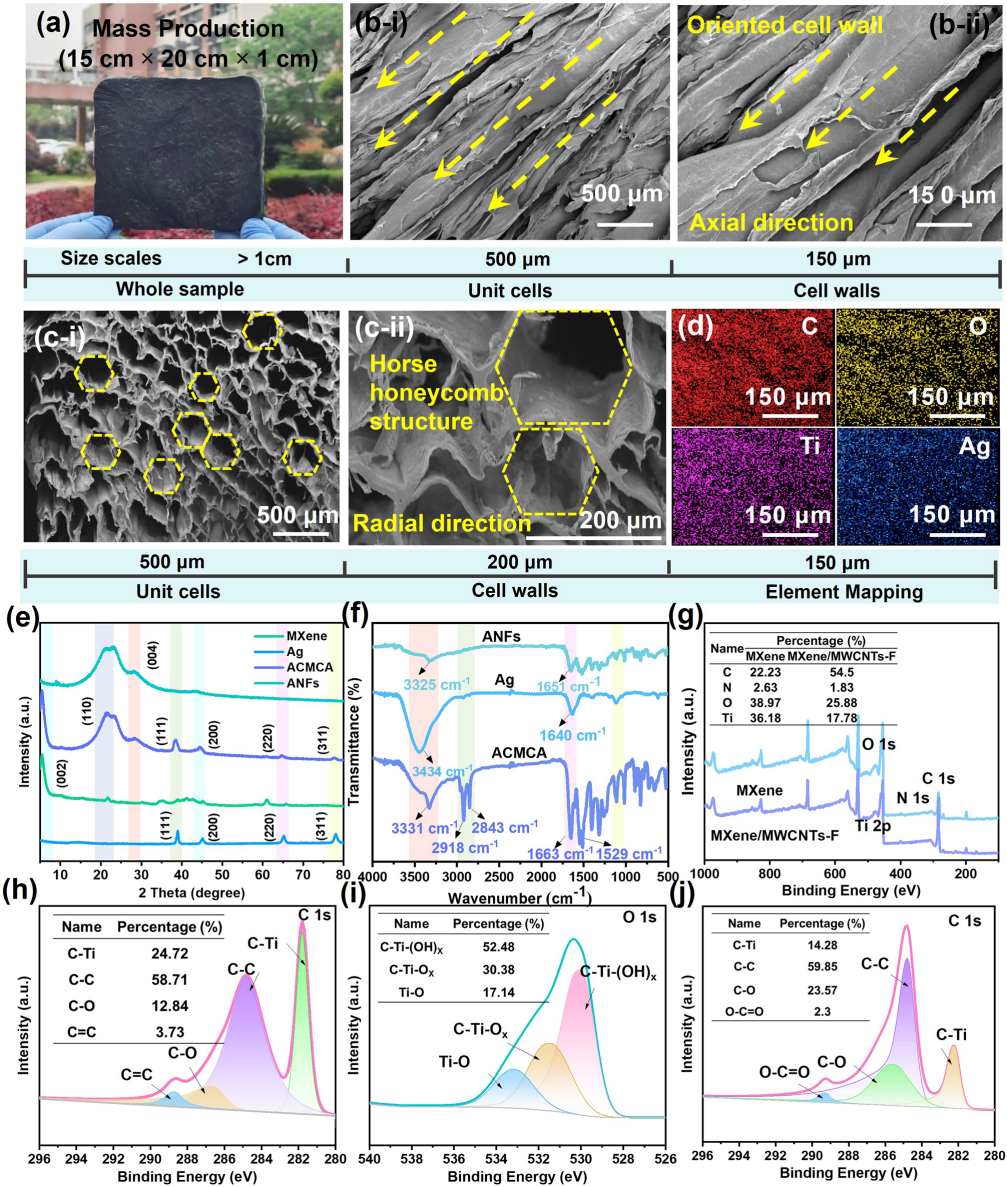
Microstructure characterization of ACMCA aerogel. **a** Large size digital photograph of ACMCA aerogel (15 cm × 20 cm × 1 cm). SEM images of **b** cross section and **c** surface honeycomb of ACMCA aerogel at **i** low and **ii** high magnification. **d** EDX profiles of different elements on the surface of ACMCA aerogel. **e** XRD patterns of MXene, Ag, ACMCA, and ANFs. **f** FTIR spectra of Ag, ACMCA and ANFs. **g** Wide-scan XPS spectra of MXene/MWCNTs-F and MXene. **h** C 1*s* and **i** O 1*s* high-resolution XPS spectra of MXene. **j** C 1*s* high-resolution XPS spectra of MXene/MWCNTs-F

The optical photographs show that ACMCA can be assembled at scale into a 3D layered porous anisotropic aerogel by utilizing a one-way ice template strategy (Fig. 2a). SEM images show that, parallel to the axis, the aerogel consisted of layered structures with large-scale parallel channels (Fig. 2b), showing a highly ordered lamellar structure with ANFs as the main skeleton. The interlayer spacing was about 20 μm, similar to the structure of human tissue muscle. The channels were connected by random bridges, and the gap between adjacent walls was about ~ 40 μm. On the axis perpendicular to the aerogel, the top view shows a honeycomb porous scaffold structure (Fig. 2c–i). The enlarged SEM images further show that the thickness of the cell wall was only a few microns, and the ANFs were intertwined to form the cell wall (Fig. 2c–ii). In addition, the EDX element mapping of the upper surface of the ACMCA aerogel showed that C (49.1 wt%), Ag (5.5 wt%), O (29.7 wt%), and Ti (15.7 wt%) elements were uniformly distributed on the surface of ACMCA. MXene, MWCNTs-F, and Ag NWs were successfully added and evenly distributed in the interior (Figs. 2d and S10, S11). The results were further verified by XRD analysis of aerogel. As shown in Fig. 2e, the typical peaks of ANFs at 20.2° and 26.9° correspond to the (110) and (004) planes, respectively, and the typical peaks of MXene at 6.1° correspond to the (002) plane. The typical peaks of Ag nanowires at 38.2°, 45.2°, 65.1°, and 78.2° correspond to (111), (200), (220), and (311) planes, respectively, whereas the typical peak of eicosane at 39.1° corresponds to (220) plane (Fig. S12). The typical peaks of ACMCA at 20.3°, 26.5°, 37.9°, and 77.6° correspond to the (110), (004), (111), and (311) planes, which proved that these substances were successfully incorporated into the aerogel. The intensity of diffraction peaks in the (200), (004), (111), and (311) planes of ACMCA decreased, mainly due to the interaction between aerogel molecules.

The existence of hydrogen bonds between aerogel molecules was further demonstrated by observing the FTIR curve of aerogel (Fig. 2f). By observing the characteristic peaks of ANFs aerogel, the stretching vibrations of C=O (1651 cm^−1^), N–H (3325 and 1532 cm^−1^), and Ph-N (1501 cm^−1^) were observed. The characteristic peaks of MXene at 3412 and 1051 cm^−1^ were the stretching vibrations of C=O and –OH, respectively (Fig. S13). The characteristic peaks of Ag NWs at 1640 and 3434 cm^−1^ were the stretching vibrations of C=O and –OH. The characteristic absorption bands of eicosane observed at 2850 and 2930 cm^−1^ were attributed primarily to the C–H bond expansion vibrations of methyl (–CH_3_) and methylene (–CH_2_–) (Fig. S14). The characteristic peaks of ACMCA were observed at 1663 (–OH), 3331 (C=O), and 2843 cm^−1^ (C–H), indicating the successful addition of these substances. Compared with ANFs aerogel, the characteristic absorption peaks of C=O and –OH of ACMCA aerogel are shifted, which further proved the above conclusion that hydrogen bonds were generated between molecules.

2D MXene nanosheets and MWCNTs-F in aerogel can be connected to each other and assembled into 3D layered structures. The chemical bond between MXene and MWCNTs-F was further analyzed by XPS test, as shown in Fig. 2g. The wide scan XPS spectra of MXene and MXene/MWCNTs-F showed peaks of C 1*s*, N 1*s*, O 1*s*, Ti 2*p*, and F 1*s*, which proved the successful preparation of ultra-thin MXene nanosheets and the successful uniform mixing of the two. The high-resolution C 1*s* spectrum of MXene showed several peaks at 281.79, 286.64, 284.8, and 288.68 eV, belonging to C–Ti, C–O, C–C, and C=C, respectively (Fig. 2h). Meanwhile, the O 1*s* spectral peak can be divided into three peaks, namely, C–Ti–(OH)_*x*_ (530 eV), C–Ti–O_*x*_ (531.42 eV), and Ti–O (533.17 eV) (Fig. 2i). The peak strength of MXene/MWCNTs-F Ti 2*p* decreased, whereas the peak strength of C 1*s* increased. The above changes in peak strength indicated the existence of an interaction force between MXene and MWCNTs-F (Fig. 2j). In addition, the peak at 457.5 eV further indicated the successful construction of a strong Ti–O–C covalent bond between MXene and MWCNs-F (Fig. S15).

### Mechanical Properties of Anisotropic ACMCA Aerogel

**Fig. 3:**
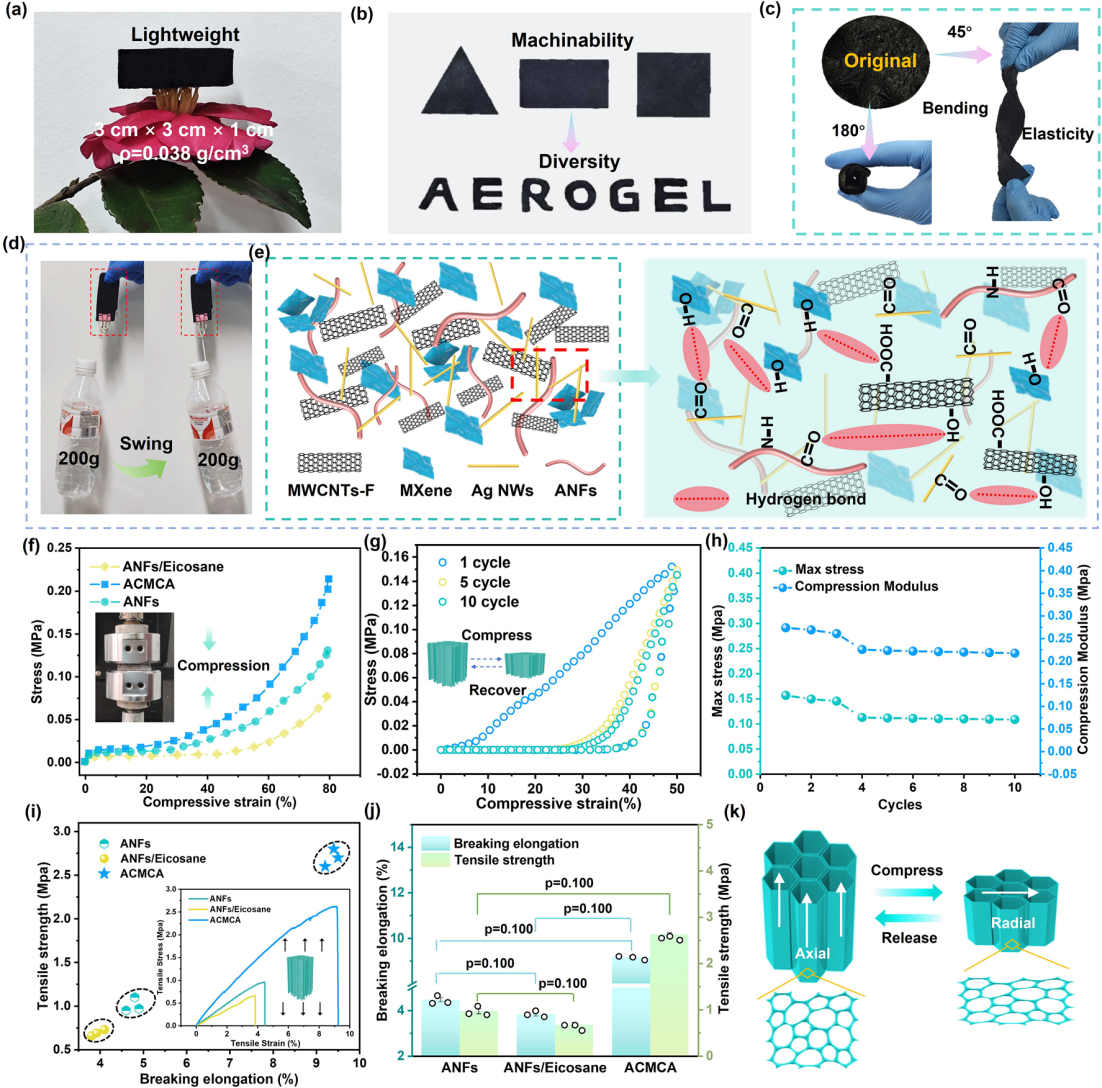
Mechanical properties of ACMCA aerogel. **a** Optical image of ACMCA aerogel standing freely on flower tips. **b** ACMCA aerogel can be cropped into a variety of digital images as required. **c** Optical images of ACMCA aerogel at 45° and 180° bends. **d** Optical images of ACMCA aerogel that can pull up a water bottle 400 times heavier than itself. **e** Schematic of the interaction force between ACMCA aerogel molecules. **f** Compressive stress–strain curves of ANFs, ANFs/eicosane, and ACMCA aerogels at 80% strain. **g** 10-cycle compressive stress–strain curve of ACMCA aerogel at 50% strain. **h** Young’s modulus and maximum stress of ACMCA aerogel at different compression cycles times.** i** Tensile stress–strain curves and scatter plots of ANF, ANFs/eicosane, and ACMCA aerogels. **j** Elongation at break and tensile strength of ANFs, ANF/eicosane, and ACMCA aerogels. **k** Schematic of stretching mechanism of ACMCA aerogel

The unique intermolecular interactions of the ACMCA aerogel not only maintained its fine pore structure in the freeze-drying process, but also endowed it with excellent mechanical properties. Figure 3a presents a digital photograph of the prepared ACMCA aerogel with regular dimensions (3 cm side length and 10 mm thickness), which can rest securely atop a flower without damaging the structure of the flower. This demonstrates that its ultra-light characteristics (low density 0.038 g cm^−3^) can better decrease the weight of firefighting clothing when used as a insulation layer and improve the rescue efficiency of firefighters. The N_2_ adsorption–desorption curves of the ACMCA aerogel displayed type IV isothermal characteristics and were accompanied by obvious hysteresis rings, indicating the presence of a large number of mesoporous. The Brunauer–Emmett–Teller results demonstrated that the ACMCA aerogel had a wide distribution of mesoporous, spanning 0.39–38 nm, with a specific surface area of up to 11.482 m^2^ g^−1^, which provided a solid foundation for low-density structures (Fig. S16). In addition, ACMCA aerogels are extremely malleable and can easily be molded into a variety of forms, including triangles, rectangles, and delicate “AEROGEL” letters, without breaking (Fig. 3b). Simultaneously, its flexibility is equally impressive, and the aerogels are folded and twisted at multiple angles of 45° and 180° without breaking (Fig. 3c). In addition, ACMCA aerogels can withstand loads 400 times their own weight, and pulling up 200 g of heavy objects can support 8 s without structural deformation and fracture, showing excellent toughness (Fig. 3d). The excellent mechanical properties of the aerogel were due to the intermolecular interactions of ANFs, MXene, MWCNTs-F, and Ag NWs. As shown in Fig. 3e, ANFs and MXene were closely linked through hydrogen bonding and chain entanglement, forming a 3D network skeleton with strong adhesion. The -OH group of MWCNTs-F formed hydrogen bonds with the C=O group of the rigid ANFs chain, and the van der Waals interaction and repulsion mode between MXene and MWCNTs-F formed uniform networks and continuous 3D short mass transfer channels, thus significantly improving the robust properties of ACMCA aerogel. It furnishes an innovative approach for the preparation of high flexibility and stretch-resistant insulation layer for firefighting clothing.

The construction of anisotropic microstructure of ACMCA, namely, the arrangement of pores, is expected to improve its compressibility and flexibility. Figure 3f shows the stress–strain curves of different aerogels under 80% compressive strain. After the introduction of phase change material eicosane, the compressive strength of ANFs/eicosane aerogel decreased to a certain extent, mainly due to the inadequate bonding at the interface between the phase change material and the aerogel, which facilitated detachment or slippage under external forces. As a result, the overall mechanical properties of the aerogel diminished. Among them, the compressive strength of ACMCA aerogel was 64% higher than that of ANFs aerogel, which further proved the existence of intermolecular interactions of aerogel. In order to further evaluate the cyclic elastic stability, we carried out 10 cyclic compression tests on the aerogel at 50% strain (Fig. 3g). The results showed that the maximum stress of ACMCA aerogel did not change significantly after five compression cycles, and remained above 70% after 10 compression cycles, which demonstrated the excellent robust and fatigue resistance properties of ACMCA aerogel. The hysteresis curve of ACMCA aerogel had three deformation stages: linear elastic deformation region associated with the elastic deformation of honeycomb cell wall (*ε* < 5%), low slope plateau region associated with the beginning of pore wall collapse (5% < *ε* < 10%), and densification with a sharp increase in stress (*ε* > 10%). As shown in Fig. 3h, the maximum compressive stress during initial compression reaches 160 kPa, and after 10 compression cycles, the maximum compressive stress and Young’s modulus barely changed. To evaluate the influence of functional materials on the robust property of ANFs-based aerogels, we tested ANFs-based aerogels by tensile test (Fig. 3i). The results displayed that the elongation at break and the tensile strength of the aerogel decreased slightly after eicosane was added. Compared with the ANFs aerogel, the elongation at break and tensile strength of ACMCA aerogel increased by 167.8% and 106%, respectively (Fig. 3j). On the one hand, the excellent mechanical properties were due to the existence of van der Waals interaction between aerogel molecules, which increases the entanglement force of the internal molecules. On the other hand, the 3D network inside the aerogel effectively buffered the impact energy and enhanced the toughness of the aerogel (Fig. 3k). Subsequently, the quasi-static compressive and tensile properties of ACMCA in the radial direction were investigated. The results showed that the axial compressive strength was higher than the radial compressive strength, and the radial elastic recovery was higher than the axial elastic recovery (Figs. S17 and S18). This difference was mainly due to the directional freezing of the aerogels, forming a lamellar structure that promotes self-support and enhances resistance to compressive stress. The radial structure can store a large compressive strain under external force, and then recover by using the stored elastic strain energy and intermolecular repulsion forces.

### Thermal Protection Properties of Anisotropic ACMCA Aerogel

**Fig. 4:**
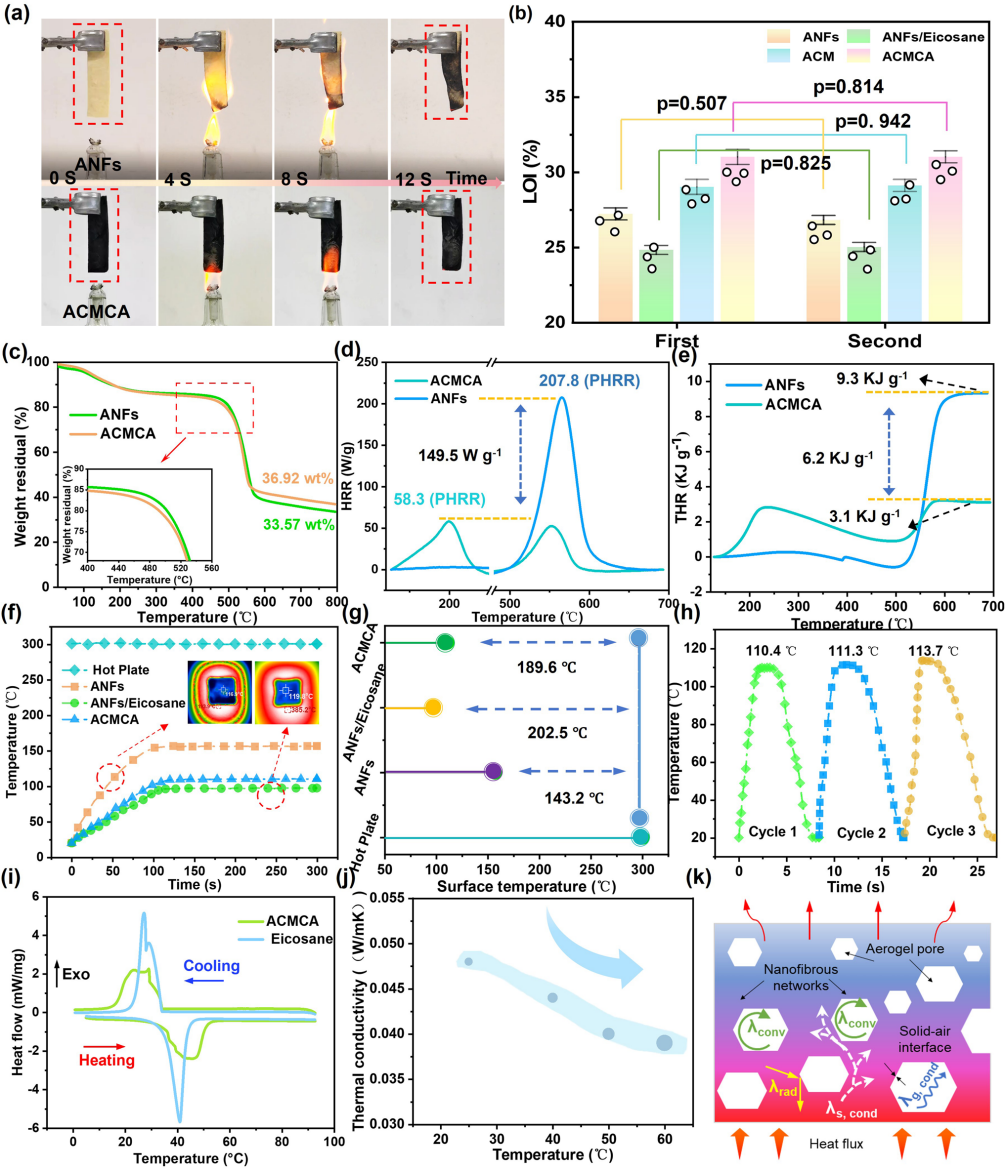
Thermal protection properties of ACMCA aerogel. **a** Alcohol lamp combustion photographs of ACMCA and ANFs aerogels (3 cm × 10 cm × 0.6 cm). **b** LOI values of ACMCA, ACM, ANFs/eicosane, and ANFs aerogels. **c** Thermogravimetric curves of ACMCA and ANFs aerogels in nitrogen atmosphere. **d** HRR curve of ANFs and ACMCA aerogels.** e** THR curve of ANFs and ACMCA aerogels. **f** Temperature–time curve of ACMCA, ANF/eicosane, and ANFs aerogels on a 300 °C heating plate. **g** Temperature difference between the equilibrium temperature of ACMCA, ANFs/eicosane, and ANFs aerogels and that of the heating plate (ΔT). **h** Temperature–time curve of heating/cooling of ACMCA aerogel circulating on the heating plate. **i** DSC curves of eicosane and ACMCA aerogel. **j** Thermal conductivity curve of ACMCA aerogel with temperature change. **k** Schematic of insulation mechanism of ACMCA aerogel

The inherent tolerance of ANFs components over a wide temperature range allows the obtained high thermoelectric nanofiber aerogels to exhibit superior resistance to extreme temperatures. This is crucial in the practical application of personal thermal protection materials, which can enable firefighters to reduce thermal damage in the surrounding environment during rescue operations. The combustion behavior of ANFs aerogel and ACMCA aerogel was further investigated using alcohol lamps (Fig. 4a). At the same time, the ANFs aerogel underwent combustion, resulting in shrinkage and deformation, accompanied with the formation of a carbon layer on the sample surface. After the alcohol lamp was removed, the ANFs aerogel quickly self-extinguished. By contrast, the ACMCA aerogel maintained original shape without any change after 12 s of combustion, and the ACMCA aerogel showed significant self-extinguishing performance after the alcohol lamp was removed (Video S1). In addition, as shown in Fig. 4b, the limiting oxygen index (LOI) of ACMCA aerogel was 31%, which was higher than that of ANFs (27%), ANFs/eicosane (24.9%), and ACM aerogel (29.1%). The LOI value decreased to some extent after the addition of phase change material. However, with the addition of MXene and other nanomaterials, the LOI value gradually increased, which can be mainly attributed to the following three aspects. First, the inherent flame retardation of MXene and other nanomaterials enables these nanomaterials to form a protective layer on the surface of the aerogel to prevent the direct contact between oxygen and the aerogel, thus delaying or preventing the combustion process. Second, the addition of nanomaterials significantly enhanced the interfacial interaction between aramid-based aerogel and phase change material. This enhanced interaction helps to form a more stable and continuous structure, reducing defects and holes that may occur during combustion, thereby improving the overall flame-retardant performance of the material. Third, nanomaterials such as MXene have excellent thermal stability, maintain structural integrity at high temperatures, and absorb and disperse heat, thereby slowing the heating rate of aerogel and reducing the risk of combustion.

To further support the accuracy and reliability of the above results, we studied the thermal decomposition behavior of aerogels and nanomaterials by thermogravimetric analysis test (Fig. 4c and Table S1). The decomposition curve of ACMCA aerogel under nitrogen atmosphere is shown as two weightlessness steps. The first weight loss occurs mainly between 100 and 200 °C, due to the evaporation of water and eicosane in the initial stage. The second weightlessness occurs between 450 and 500 °C, with a weight loss of about 63%. The significant weight loss in this temperature range was mainly due to the thermal decomposition of ANFs skeleton, MXene, and eicosane, as well as the thermal decomposition of C=O, N–H, and other functional groups. The decomposition curves of ANFs aerogel, MXene, and eicosane could prove the above results, and eicosane has a weight loss of 99% at 210 °C (Fig. S19). FTIR and XRD tests were used to study the chemical and microstructure changes of ACMCA after combustion. The data displayed that the characteristic peaks of N–H, C = O, and –OH groups disappeared after combustion. In XRD test, after combustion, the (002) crystal plane disappeared, and the (110), (101) crystal planes appeared. The thermal degradation of functional groups and ANFs skeleton and the formation of titanium dioxide (TiO_2_) were further demonstrated (Fig. S20). In addition, TG-FTIR spectroscopy was performed on ACMCA aerogel to further study whether ACMCA releases toxic gases during combustion (Fig. S21). The results showed that water vapor (1670, 2500, and 3450 cm^−1^) and carbon dioxide (CO_2_, 2350 cm^−1^) were generated during combustion of the aerogel. No toxic gases were produced.

In addition, in order to simulate the burning behavior of firefighting clothing in real situations during rescue operations, the flame retardancy of aerogel materials was tested by micro-calorimetry (Fig. 4d, e). The peak heat release rate (PHRR) values of ACMCA and ANFs aerogels were 58.3 and 207.8 W g^−1^, respectively, and the total heat release rate (THR) values were 3.1 and 9.3 kJ g^−1^. Compared with ANFs aerogel, the PHRR and THR values of ACMCA aerogel were reduced by 80% and 45%, respectively, which further proved that the designed aramid-based aerogel had excellent flame retardation. At the same time, SEM images of the ACMCA aerogel after combustion showed that a dense carbon layer formed on the upper surface of the aerogel, the cell wall became thin, the layered structure became loose, and the layer spacing increased (Fig. S22). On the basis of the above studies, we proposed the flame-retardant mechanism of ACMCA aerogel (Fig. S23). In the combustion process, MXene reacts with oxygen to form a solid TiO_2_ carbon layer, which inhibits the thermal decomposition and oxygen erosion of the ACMCA aerogel. At the same time, MWCNTs-F and ANFs undergo thermal decomposition at elevated temperatures, releasing nonflammable gases (CO_2_, CO, N_2_, and H_2_O), which reduced oxygen concentration and inhibited the combustion reaction. In addition, MXene lamellae can form physical barriers inside the aerogel like a “brick wall,” blocking the contact between oxygen and the aerogel matrix, thus inhibiting the combustion reaction. At the same time, MXene lamella can also prevent the outward diffusion of combustible gas generated during the combustion of aerogel, reduce the concentration of combustible gas in confined space, reduce the contact opportunity with oxygen, and thus reduce the intensity of combustion. The excellent flame-retardant properties make ACMCA aerogel have good conditions for the application in the field of heat insulation barrier of firefighting clothing.

To better realize the application of ANFs-based composite aerogel in personal thermal protection, the excellent thermal insulation performance of aerogel is also essential. Incorporating a typical negative temperature coefficient thermal switching phase change material (eicosane) into ANFs-based aerogels effectively restrained the heat transfer, increased the temperature difference between the two ends, and significantly reduced the thermal conductivity of the aerogels. In order to verify this conclusion, we fixed the heating plate at a constant temperature of 300 °C, and recorded the temperature changes in the equilibrium process of the aerogel (ANFs, ANFs/eicosane, and ACMCA) with an infrared thermal imager (Fig. S24). Figure 4f shows the temperature–time curve corresponding to the dynamic equilibrium process of the aerogel. After a period of time, the upper surface temperature of the aerogels reached a certain temperature and did not change. The upper surface equilibrium temperature of ANFs, ACMCA, and ANFs/eicosane was 156.8, 110.4, and 97.5 °C, respectively, and the temperature difference (ΔT) with the heating plate was 143.2, 189.6, and 202.5 °C, respectively (Fig. 4g). The above data showed that the addition of eicosane significantly enhanced the scattering effect during heat transfer, effectively inhibited heat transfer, and enhanced the thermal insulation ability of aerogel. Subsequently, ACMCA aerogel was subjected to heating (25–300 °C) and cooling (300–25 °C) cycles to test its cyclic temperature regulation performance. After eight cycles, the equilibrium temperature of the ACMCA aerogel hardly changed (Figs. 4h and S25). The compatibility between eicosane and ANFs-based aerogel was studied by DSC curve. The results showed that no new peaks appeared, and the compatibility between eicosane and ANFs-based aerogel was good. Moreover, Tpm (melting temperature) and Tom (melting onset temperature) of ACMCA aerogel were about 45.7 and 35.6 °C, respectively, and these values were close to that of eicosane. This indicated that the endothermic peak of ACMCA was caused by the melting of eicosane (Fig. 4i and Table S2). In addition, studies on eicosane leak experiments were performed on eicosane and ACMCA aerogel for 60 s (Fig. S26). As a result of the 3D network structure of ACMCA aerogel and the strong capillary force, eicosane was completely converted to a liquid state by heating, while the ACMCA aerogel retained its initial form without eicosane leakage. These results demonstrated the great potential of ACMCA aerogel application in long-term thermal regulation of firefighting clothing, and excellent thermal insulation performance can reduce the heat penetration of firefighting clothing. Figure 4j shows the temperature thermal conductivity curve of ACMCA aerogel with temperature change. As the temperature increased, the thermal conductivity of the aerogel decreases significantly. The main reason was that when the temperature increased, the molecular spacing of eicosane increased, the arrangement became loose, and the molecular movement became more violent and disorderly, which impeded the orderly transfer of heat and resulted in the decrease of thermal conductivity. This characteristic enables aerogels to possess extensive application prospect in temperature control.

In order to evaluate the properties of ANFs aerogel under extremely cold environment, the aerogel was placed on an ice plate at − 20 °C. After 30 s, the upper surface temperature of the aerogel hardly changed, showing excellent insulation effect (Fig. S27). Compared with the typical thermal insulation materials studied at present, the obtained ACMCA aerogel performed well in terms of light weight and low thermal conductivity (Figs. S28 and S29). Subsequently, the ACMCA aerogel was placed in liquid nitrogen at a temperature of − 196 °C for 3 min. After a long period of freezing, the ACMCA aerogel could still bend, demonstrating its remarkable flexibility and mechanical robustness under cold conditions (Fig. S30). Based on the above studies, we propose the mechanism by which the ACMCA aerogel inhibited heat transfer behavior. As shown in Fig. 4k, the effective thermal conductivity of porous aerogel (*λ*_eff_) was theoretically determined by the thermal conductivity of solid (*λ*_s, cond_), gas (*λ*_g, cond_), thermal convection (*λ*_conv_), and thermal radiation (*λ*_rad_). Of these, *λ*_conv_ is usually negligible. The numerous solid–air interfaces enhances radiation reflectivity, and *λ*_rad_ can be effectively reduced. The high porosity and low-density structure effectively increased the interface thermal contact resistance (i.e., Kapitza resistance) and reduced the decrease of *λ*_s, cond_. *λ*_g, cond_ can be estimated using Eq. 1:

1 $$ \lambda_{{g,{\text{cond}}}} = \frac{{\lambda_{g0} }}{1 + 2\alpha l/D} $$

where *α* = 2 of air, λ_g0_ = 26 mW m^−1^ K^−1^, *D* is the average pore diameter of the material, and *l* is the average free path of air molecules. Compared with traditional porous materials (Fig. S31), the ACMCA aerogel had a smaller pore size (18.205 nm), which effectively blocked the pathway of phonons transport, restricted the conduction of solids, and significantly reduced *λ*_*g*, cond_.

### Thermogenic Temperature Sensing of Anisotropic ACMCA Aerogel

Thermoelectric power generation can directly convert heat into voltage, enabling temperature sensing by monitoring changes in voltage signals in the absence of an external power source. Inspired by the oriented structure of human muscle tissue, the ACMCA aerogel prepared by directional freezing of liquid nitrogen has enhanced temperature sensing property in the axial direction. To verify this view, we subjected non-ACMCA (non-anisotropic ACMCA) and ACMCA aerogels to heating on a heating plate at 400 °C, and the output voltage of aerogels was recorded (Fig. S32). Under the same heating conditions, the output voltage of ACMCA aerogel could reach 16.15 mV, but the non-ACMCA aerogel required 40 s to reach the maximum output voltage of 10.75 mV. After the construction of the ordered structure, the output voltage of aerogel increased by 50.2%. The optimization of the directional ordered structure positively influenced the ability of the aerogels to percept temperature with high efficiency, as further confirmed by the above results.

**Fig. 5:**
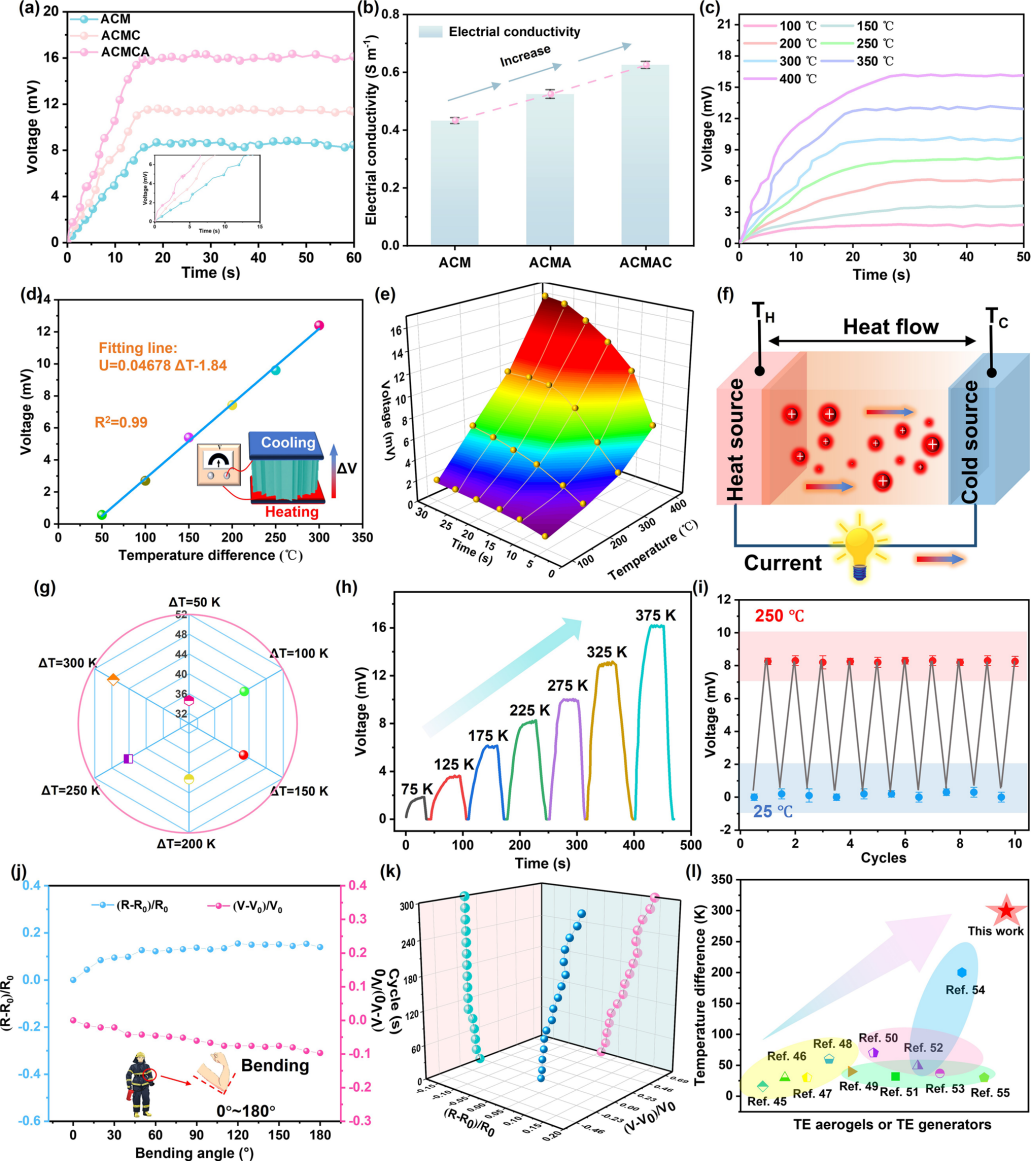
Temperature sensing properties of ACMCA aerogel. **a** Time output voltage curve of ANFs, ACMC, and ACMCA aerogels under the action of alcohol flame. **b** Conductivity of ANFs, ACMC, and ACMCA aerogels. **c** Output voltage curves of ACMCA aerogel at different temperatures. **d** Linear fitting curve of output voltage and temperature corresponding to ACMCA aerogel. **e** 3D output voltage data diagram of ACMCA aerogel at different heating temperatures from 100 to 400 °C. **f** Schematic of the temperature sensing mechanism of ACMCA aerogel. **g** Seebeck coefficient of composite aerogel at different temperature differences. **h** Voltage sensitivity curve of ACMCA aerogel at different temperatures. **i** Output voltage curve of ACMCA aerogel during alternating heating (250 °C) and cooling (room temperature) for 10 cycles. **j** Curve of voltage change rate and resistance change rate of ACMCA aerogel at different bending angles of 0°–180°. **k** Curve of voltage change rate and resistance change rate of ACMCA aerogel after 300 cycles at different bending angles of 0°–180°. **l** Comparison with the maximum temperature sensing temperature of other thermoelectric aerogels on the market [45–55]

In order to further explore the thermoelectric conversion performance of ANFs-based aerogel under real fire conditions, the combustion test of ANFs-based aerogels was carried out with an alcohol lamp, and the time–voltage curve changes of the aerogel in the combustion process of alcohol lamp were recorded in detail (Fig. 5a). Under the same combustion conditions, the output voltage of ACMAC was the highest, which could reach 16.1 mV, and the performance was the most prominent among all aerogels. The reason was that the van der Waals interaction between MXene nanosheets and MWCNTs-F promoted the construction of 3D uniform network architecture and conductive channels, and the integration of Ag NWs further accelerated the carrier transmission rate, which significantly improved the thermoelectric conversion efficiency. As shown in Fig. 5b, the conductivity of ACMCA aerogel was 0.67 S m^−1^, which was 0.2 S m^−1^ higher than that of ACM aerogel, which also provided strong evidence for the above conclusion. Figure 5c shows the output voltage variation of ACMCA thermoelectric aerogel when heated at different temperatures (100–400 °C). When the heating temperature increased from 100 to 400 °C, the output voltage of ACMCA thermoelectric aerogel gradually increased from 1.4 to 16.7 mV in 26 s (an increase of 15.3 mV).

Under varying temperature difference ranges, the maximum output voltage of ACMCA aerogel was analyzed, resulting in a linear fitting curve correlating temperature difference and output voltage. The fitting function formula was expressed as *U*_max_ = 0.04678*ΔT-*1.84, and the linear fitting degree *R*^2^ was 0.99. The temperature difference is proportional to the output voltage of the ACMCA aerogel, and the greater the temperature difference, the higher the output voltage. When Δ*T* = 300 K, The maximum output voltage of ACMCA was stable at 12.3 mV, close to the theoretical value of 12.194 mV calculated by the formula *U*_max_ = 0.04678*ΔT-*1.84, showing the high accuracy of temperature sensing (Fig. 5d). Under the same experimental conditions, the maximum output voltage of non-ACMCA aerogel was fitted, and the fitting function formula *U*_max_ = 0.0325*ΔT-*0.46 was obtained, the linear fitting *R*^2^ was 0.97, and Seebeck coefficient was lower than the value of ACMCA aerogel (Fig. S33). The increase in Seebeck’s coefficient allows ACMCA aerogels to accurately sense minor temperature changes, improving the accuracy of temperature measurements. Therefore, by analyzing the relationship between the output voltage and the temperature difference, the surface temperature of the insulation layer of firefighting clothing can be accurately determined, so as to trigger the high-temperature warning threshold accurately and sensitively, and reduce the possibility of the firefighters’ lives being harmed. As a result of the Seebeck Effect, the thermoelectric conversion efficiency of ACMCA aerogel also increased with the increase in heating temperature (Fig. 5e). Due to the temperature difference between the upper and lower ends of the aerogel, charge carriers migrated in a directional way from the high-temperature region to the low-temperature region along the temperature gradient inside the aerogel, generating a potential difference at both ends of the thermoelectric aerogel, that is, the output voltage (Fig. 5f). The electromotive force is proportional to the temperature difference, the greater the temperature difference, the more intense the directional motion of the charge carrier inside the thermoelectric aerogel, and the more energy the temperature difference provides. Thus, the charge carrier can easily overcome the potential barrier inside the material to achieve directional movement. The Seebeck coefficient of ACMCA aerogel gradually increased with the increase of temperature difference, mainly because the mobility of carrier inside ACMCA aerogel increased with the increase in temperature difference (Fig. 5g).

As the external temperature difference (ΔT) stimulus applied to the aerogel increased from 75 to 375 K, the real-time voltage increases linearly, confirming that the aerogel sensor could accurately detect temperature changes with excellent sensitivity (Fig. 5h). More importantly, the aerogel sensor also exhibited excellent sensing stability and repeatability, which could be demonstrated by the voltage response to steady-state and alternating temperature differences. The ACMCA aerogels were circulated in the range of 25–250 °C for 10 cycles of heating–cooling (room temperature) operation (Fig. 5i). After 10 repeated heating and cooling cycles, the maximum output voltage was stable at about 8.13 mV, which was close to the theoretical value of 8.23 mV calculated by the formula *U*_max_ = 0.04678*ΔT-*1.84. In addition, the changes in (*R*-*R*_0_)/*R*_0_ (*R*_0_ and *R* are the resistance before and after bending, respectively) and (*V*-*V*_0_)/*V*_0_ (*V* and *V*_0_ are the output voltage before and after bending, respectively) at room temperature under different bending angles (0°–180°) were studied (Fig. 5j). The results showed that the variation range of resistance was less than 15.5%, and the maximum loss rate of output voltage was 9.6%. To further demonstrate the durability of ACMCA aerogels, we measured the change in (*R*-*R*_0_)/*R*_*0*_ and (*V*-*V*_0_)/*V*_0_ after 300 bending and folding cycles (Fig. 5k). The results showed that the maximum fluctuation of voltage and resistance was 13.0% and 13.5%, respectively, after 300 bending and folding cycles. We tested the durability of ACMCA aerogel under different washing times and twisting times at room temperature (Fig. S34). The results showed that after multiple washings and twisting, the range of resistance changes was less than 9.4% and 7.15%, respectively. They are further proved that ACMCA aerogel had stable and reversible temperature sensing properties, which could be used to accurately and timely monitor the early temperature rise of firefighting clothing in a fire. The ACMCA aerogel exhibited excellent TE detection sensitivity and TE stability mainly due to the following two aspects. ANFs, as a long-chain skeleton supporting the aerogel, could withstand large mechanical deformation and the interaction between internal molecules, further improving the mechanical stability of the aerogel. The oriented structure of the aerogel and the synergistic effect of the highly conductive pathways of MXene, Ag NWs, and MWCNTs-F rendered the internal structure of the ACMCA aerogel robust and very stable. Compared with the highest test temperature of most TE aerogels and their generators reported in the current literature, the test or application temperature of the ACMCA aerogel developed in this study is significantly higher, which further proves its excellent temperature resistance and efficient thermoelectric conversion ability. It showed that it had good adaptability to various temperature conditions and had great prospects in the application field of firefighting clothing (Fig. 5l).

### High Temperature and NH_3_ Warning Performance of Anisotropic ACMCA Aerogel

**Fig. 6:**
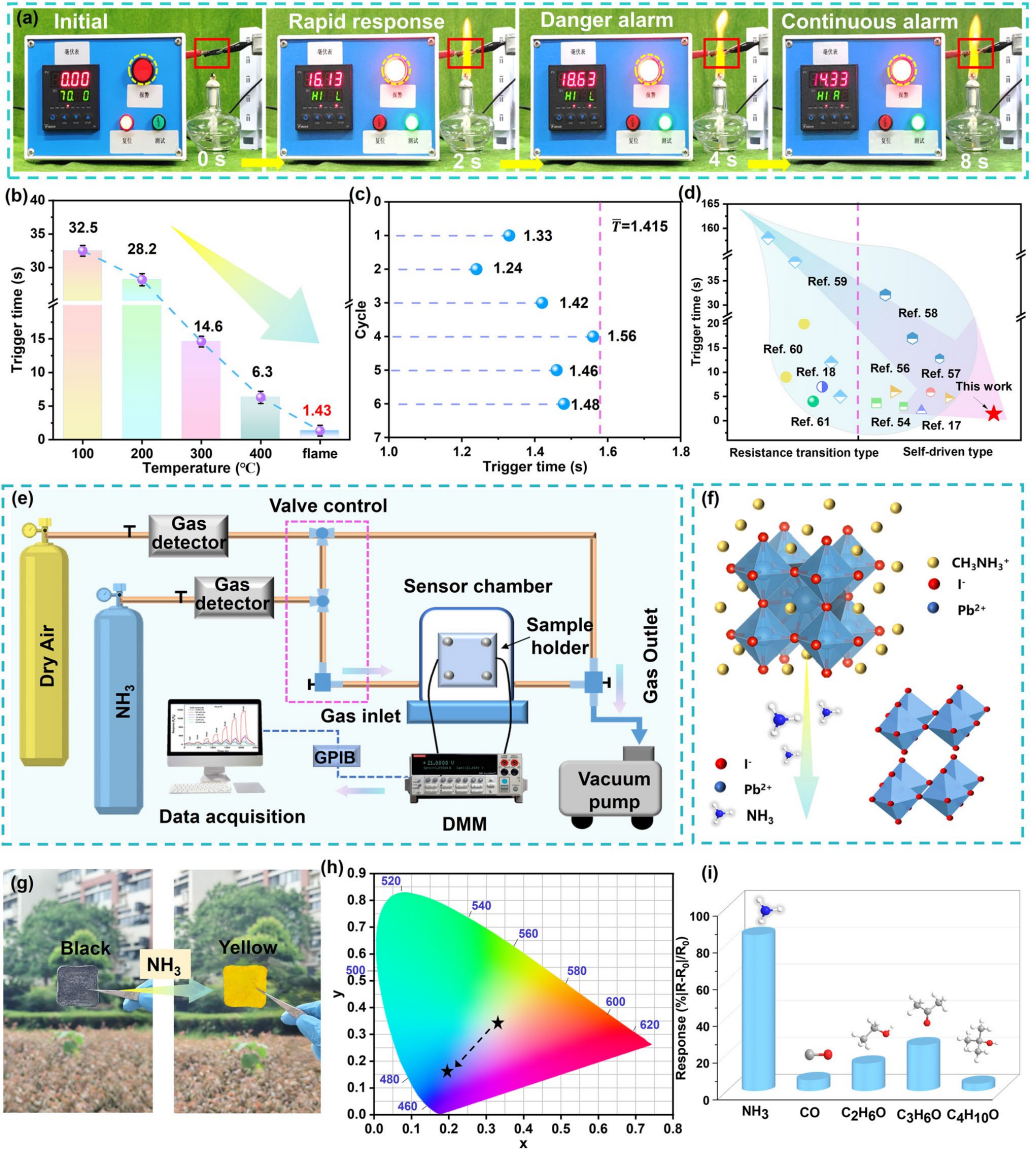
High-temperature warning performance of ACMCA aerogel. **a** High-temperature warning test of ACMCA aerogel when exposed to alcohol lamp flame. **b** Trigger time of ACMCA aerogel alarm at different temperatures. **c** Trigger time during six alarms cycles of ACMCA aerogel. **d** High-temperature alarm response time of self-powered ACMCA aerogel compared with other reported sensor materials, including the resistance transition and self-driven alarm types [17, 18, 54, 56–61]. **e** Schematic diagram of gas monitoring device for ACMCA-M aerogel. **f** Schematic of ACMCA-M aerogel gas monitoring. **g** Optical image of color change after monitoring ammonia gas by ACMCA-M aerogel. **h** CIE 1931 chromaticity diagram of ACMCA-M aerogel. **i** Gas monitoring selective comparison of ACMCA-M aerogel to other gas, including the detection of NH_3_, CO, C_2_H_6_O, C_3_H_6_O, C_4_H_10_O

Inspired by the oriented structure of muscle tissue, the resultant ACMCA aerogel exhibited high-efficiency thermoelectric conversion property, which effectively improved the sensitivity of high-temperature warning. In this study, ACMCA and non-ACMCA (non-anisotropic ACMCA) aerogels were connected with a millivolt alarm, respectively, and 1 mV was established as the early warning trigger voltage to construct a high-temperature alarm system and assess its effectiveness. After contact with high flame temperature, the output voltage of ACMCA and non-ACMCA aerogel begins to rise, and when it exceeds 1 mV, the high-temperature warning system is triggered. By establishing a warning trigger voltage of 1 mV, the ACMCA aerogel can trigger the alarm within 1.5 s of contact with a flame, whereas the non-ACMCA aerogel requires 5.2 s. This results further confirmed that the directional structure of ACMCA enhanced itself high-temperature warning sensitivity (Figs. 6a and S35). In order to monitor the temperature recognition ability of ACMCA at different temperatures, we tested the trigger time of its high-temperature alarm when the samples were put on the heating plate (Fig. 6b). As temperature increased, the high-temperature warning time decreased significantly (32.5 s at 100 °C, 28.2 s at 200 °C, 14.6 s at 300 °C, 6.3 s at 400 °C, and 1.43 s at alcohol lamp flame), demonstrating the sensitive response ability to temperature. The reversible TE response property of the ACMCA aerogel enables the high-temperature alarm system to be activated multiple times when the ACMCA encounters a flame again, offering a solution to the defect of nonreusable Go-based high-temperature alarm sensors. As shown in Fig. S36, ACMCA was intermittently exposed to the flame for 20 s with an average output voltage of 8.04 mV, demonstrating excellent repeatability. Figure S37 is a screenshot of a video of ACMCA aerogels repeating the high-temperature alarm test when exposed to an alcohol lamp. When the alcohol lamp flame was encountered again, the ACMCA aerogel could reactivate the alarm light within 1.5 s (Video S2). After five cycles, the ACMCA aerogel still had an ultra-sensitive high-temperature warning performance of 1.48 s. In the six fire alarm cycles, the response times of the first and fifth cycles were 1.33 and 1.46 s, respectively, confirming the stability and durability of the high-temperature alarm performance (Fig. 6c). Overall, the ACMCA aerogels exhibited sensitive alarm response times and repeated alarm capabilities, which could promptly alert firefighters before the protective becomes damaged in the extreme high-temperature environment. According to the above results of ACMCA aerogel, we further compared the high-temperature alarm response time at different elevated temperatures between our work and various other studies, including the resistance transition and self-driven alarm types (Fig. 6d). The results of the comprehensive comparison showed that the ACMCA aerogel exhibited the shortest response time of 1.43 s without depend on external batteries for power based on the mechanism of directly converts heat into electrical voltage to trigger warning without the need for an external power supply.

In the harsh fire environment, especially in some industrial sites, there may be leakage of toxic gas (NH_3_). Firefighters exposed to high levels of NH_3_ for long time periods not only can cause serious human effects such as eye, throat, skin, and respiratory irritation but also tend to cause explosions when exceeding a certain concentration. However, NH_3_ is colorless and rapidly diffused, which is difficult to detect with the naked eye, so the ability to visually monitor NH_3_ is essential to ensure the safety of firefighters. In this study, CH_3_NH_3_PBI_3_ (MAPbI_3_) was combined into anisotropic ACMCA to produce a novel composite aerogel (ACMCA-M) for visual monitoring of NH_3_ in complex fire situations, which solved the limitation of traditional high-temperature warning sensors lacking visually waning function of dangerous gases. We constructed a digital gas-sensitive measurement system using a digital multimeter (DMM) to monitor resistance of ACMCA-M aerogel, and collected the data in real time by a data acquisition system (Fig. 6e). ACMCA-M aerogel was fixed in the chamber and blown with air, and then dry air passed through the chamber, recording its baseline resistance. Subsequently, a 40 ppm concentration of NH_3_ gas was injected for cyclic testing at room temperature, revealing that ACMCA-M aerogels possessed reversible NH_3_ detection function with a sensitivity reached 87.3% (Fig. S38). The ACMCA-M aerogel showed high selectivity and sensitivity to NH_3_, mainly due to the structural transformation of MAPbI_3_ and its decomposition into PbI_2_ when exposed to NH_3_ (Fig. 6f). When exposed to NH_3_, the appearance of ACMCA-M aerogel underwent a visual shift from black to yellow, further demonstrating the generation of PbI_2_ (Fig. 6g). The CIE 1931 chromaticity diagram also illustrated the discoloration process of ACMCA-M aerogel from black to yellow during NH_3_ monitoring (Fig. 6h). In addition, we further used a gas-sensitive characterization system to monitor different gases (e.g., C_2_H_6_O, CO, C_3_H_6_O, C_4_H_10_O, and NH_3_) at the same concentration (40 ppm), and the results proved that the ACMCA-M aerogel had good selectivity for NH_3_ (Fig. 6i). The visual monitoring of NH_3_ gas can provide an intuitive and rapid response, increasing firefighters’ risk awareness in challenging fire environments during fire operations and rescue.

### Extended Application of ACMCA in Firefighting Clothing

**Fig. 7:**
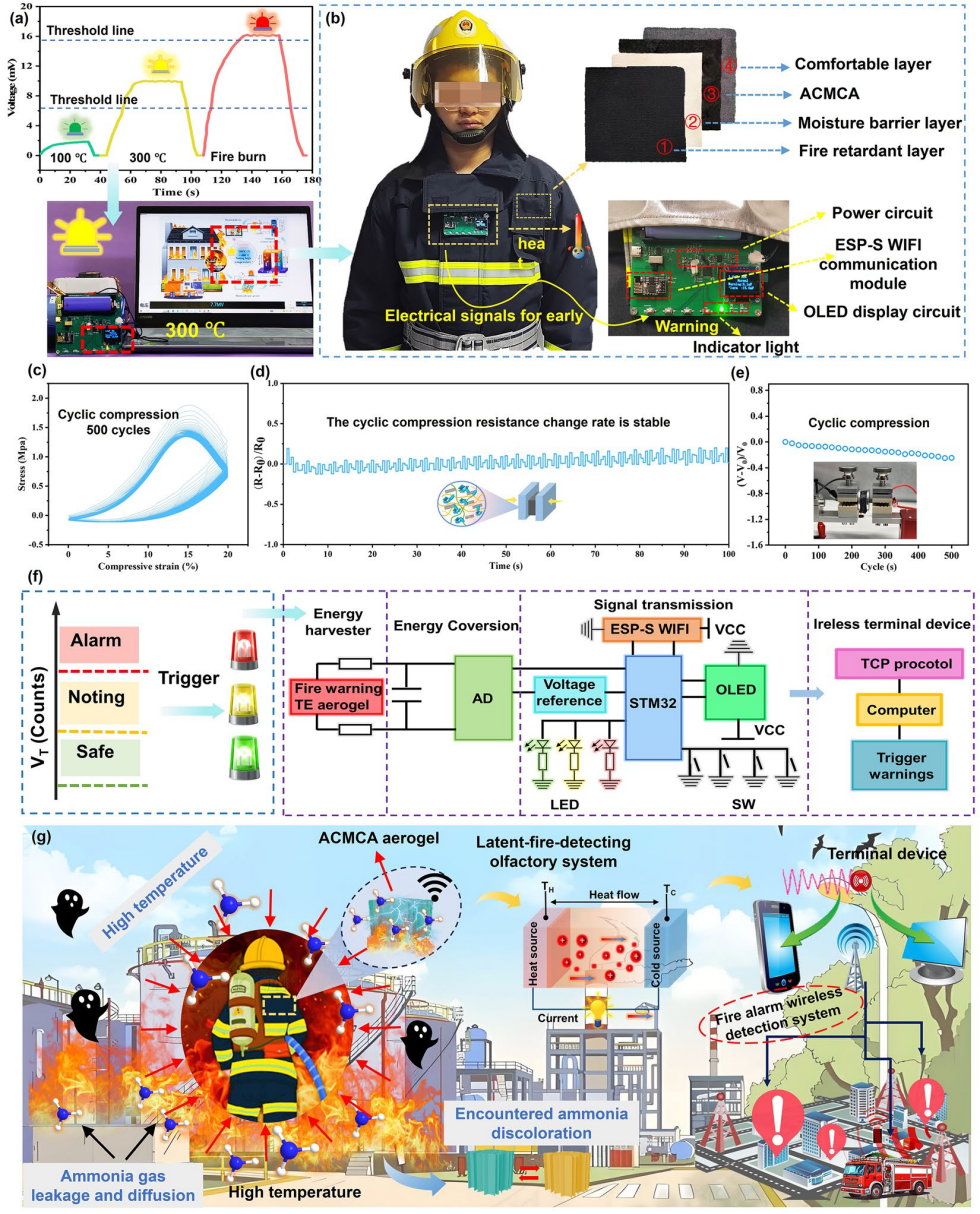
Extended application of ACMCA-based high-temperature alarm system in firefighting clothing. **a** Multistage high-temperature alarm test of ACMCA aerogel on a heating plate at 300 °C. **b** Schematic of the application of ACMCA aerogel in the insulation layer of firefighting clothing for classification early warning. **c** Compressive stress–strain curve, **d** resistance change, **e** TE output voltage changes of ACMCA aerogel over 500 compression cycles at 20% strain. **f** Electronic design automation schematic diagram of multistage high-temperature alarm system. **g** Extended application diagram of ACMCA-based multistage wireless high-temperature alarm system when facing the complex fire environment involving the high temperature and explosive gas in fire cases

On the basis of the high-efficiency TE conversion and precise temperature perceptual capabilities of the prepared ACMCA aerogel, we combined it with a developed multistage wireless high-temperature alarm system into firefighting clothing. The developed graded and self-driven multistage wireless alarm system with a size of (3 cm × 3 cm × 1 cm), which can be integrated into the firefighting protective clothing as well as the ACMCA. The ACMCA-based self-driven high-temperature alarm system can achieve accurate high-temperature monitoring for firefighting clothing before protective clothing thermal damage in fire. In this temperature monitoring and early warning system, we established predetermined thresholds for high and low temperatures, using three different colors of LED lights as indicators for “Safe” state, “Noting high temperature” state, and “Warning” state of firefighting clothing. To verify the precise high-temperature warning function, the temperature sensing ACMCA aerogel was fixed on the temperature control platform and connected with the wireless alarm system to trigger the warning through TE generation of ACMCA. The Alert Status threshold of the second-level warning system was set to 200 °C, and the Danger Alarm Status threshold of the third-level warning system was set to 400 °C, corresponding to the output voltage thresholds of 6.8 and 15.6 mV, respectively. To verify the accuracy of ACMCA aerogel classification warning, we heated the TE aerogel at different temperatures. In the absence of temperature, everything remained calm. However, as soon as the temperature was detected, the early warning system was quickly activated and showed different levels of warning (Fig. 7a). As shown in Fig. S39, when the aerogel was below 200 °C, the green light prompt was triggered, indicating a “Safe state” (first-level warning). Between 200 and 400 °C, the yellow light prompt was triggered, indicating a “Noting high temperature” state (second-level warning). When the temperature exceeded 400 °C, the red light was triggered (third-level warning), and the “warning transform state” was displayed by Video S3. The application of ACMCA as an enhanced insulation layer in firefighting clothing not only replaces the cumbersome feeling associated with conventional thermal insulation but also introduces a significant advancement in high-temperature warning capabilities in firefighting protective clothing (Fig. 7b). When firefighters carry out rescue operations, a sensitive and accurate alarm system in firefighting clothing can ensure the safety of firefighters in fire cases. Moreover, the intelligent self-damage perception capability of firefighting clothing assists firefighters in implementing varying rescue strategies to avoid the failure of the rescue mission caused by damage to firefighting clothing in extreme high-temperature situations.

In order to simulate the durability performance of ACMCA during real wearing process in firefighting clothing, we conducted a cyclic compression test for the electrical conductivity of ACMCA. Figure 7c shows the compressive stress–strain curve of ACMCA under 500 cycles of compression at a strain of 20%. Results reveal that the ACMCA exhibits stable resistance to compression and super fatigue resistance due to their anisotropic structures. Figures 7d, e and S40 show the electrical resistance change rate (*R-R*_*0*_*/R*_*0*_) and TE output voltage change rate (*V-V*_*0*_*/V*_*0*_) during the identical cyclic compression process. After 500 times of compression, the resistance change rate and voltage change rate of ACMCA only changed slightly, demonstrating excellent electrical related stability. In addition, we also added 10% compression strain and 10 cycles of resistance change test, which further proved the excellent stability of ACMCA aerogel (Fig. S41). Moreover, Fig. 7f further depicts the multistage warning data diagram and logical design of the high-temperature alarm system. The electronic design automation schematic diagram of alarm system reveals that the warning system mainly includes voltage acquisition and processing, display circuit, different grade indicator light, calibration button, communication module, voltage reference, power circuit, STM32 microcontroller unit, and other modules (Fig. S42). The ACMCA aerogel will generate a TE voltage signal at high temperatures, which is collected by the microcontroller to reflect temperature variations. When the voltage signal exceeds the warning trigger threshold, the wireless transmitter will send instructions to the terminal receiver (Fig. S43). Accordingly, the wireless signal activates the indicator light and the firefighters can quickly understand the safety status of protective clothing based on the color of indicator light. The above results show that the multistage wireless alarm system based on ACMCA possesses promising application prospects for real-time temperature monitoring and multistage high-temperature warning ability. Figure 7g depicts the extended application of ACMCA or ACMCA-M aerogel to firefighting clothing, which can be achieved ultra-sensitive temperature and visual toxic gas monitoring in complex fire environment. In high-temperature environments, aerogel can monitor the temperature of the insulation layer surface in real time based on the linear relationship between temperature difference and voltage. When the output voltage exceeds the set threshold, the wireless alarm system sends an early warning signal to the terminal through Bluetooth transmission. The activated indicator light in firefighting clothing or received danger signal by fire base station can remind firefighters to evacuate in time before their life safety is compromised. Overall, inspired by the human muscle, the resulting anisotropic TE aerogel is able to ensure the operation safety of firefighters when facing the complex fire environment involving the high-temperature even flammable and explosive gas in fire cases. In addition, we have a deeper understanding of the potential future research of ACMCA aerogel. For example, ACMCA aerogel health detection function can be given to real-time analysis of the wearer’s blood sugar, pulse, and other early warning before illness, so as to further improve the life safety of the wearer.

## Conclusion

In summary, inspired by the human muscle, an anisotropic fire safety aerogel ACMCA with precise self-temperature perceptual function is developed by combining aramid nanofibers with eicosane/MXene to form an anisotropically oriented conductive network. Benefiting from the anisotropic structural configuration, the resulting ACMCA exhibited exceptional mechanical properties (tensile strength of 2.52 MPa and compressive strength of 0.21 MPa) and direction-dependent electrical conductivity (perpendicular direction 0.625 S m^−1^). By combining the negative temperature-dependent thermal conductivity of C20 with the highly ordered conductive network of MXene formed along the directional freezing axis, the ACMCA exhibited remarkable thermoelectric properties, achieving S values of up to 46.78 μV K^−1^ at room temperature. Moreover, the ACMCA aerogel possessed an ultra-light density of 0.038 g cm^−3^ and ultra-low thermal conductivity of 0.048 W m^−1^ K^−1^, indicating its practical application in firefighting clothing. Moreover, the ACMCA aerogel utilized the thermoelectric effect to convert heat into electrical voltage, displaying a real-time linear voltage response to temperature variations. The combination of the thermoelectric effect of ACMCA with a self-developed wireless high-temperature warning system demonstrated a wide temperature sensing range (50–400 °C) and a rapid warning response time to extreme temperatures (~ 1.43 s), enabling timely alerts for firefighters to evacuate. The anisotropic aerogel for temperature sensing described in this work offers a novel approach to the design of next-generation thermal barrier layers for firefighting clothing, thereby enhancing firefighter safety during operations.

## Supplementary Information

Below is the link to the electronic supplementary material.

Supplementary file1 (MP4 3176 KB)

Supplementary file2 (MP4 13270 KB)

Supplementary file3 (MP4 3742 KB)

Supplementary file4 (DOCX 12708 KB)
