# Supplementary material for: Muscle-Inspired Anisotropic Aramid Nanofibers Aerogel Exhibiting High-Efficiency Thermoelectric Conversion and Precise Temperature Monitoring for Firefighting Clothing
Source: Nanomicro Lett. 2025 Apr 14;17:214. doi: 10.1007/s40820-025-01728-x (PMC11996746; doi:10.1007/s40820-025-01728-x)
Supplement: Supplementary file 4 — Supplementary file4 (DOCX 12708 KB) [file 40820_2025_1728_MOESM4_ESM.docx]

Supporting Information for

**Muscle-Inspired Anisotropic Aramid Nanofibers Aerogel Exhibiting High** **Efficiency Thermoelectric** **Conversion and Precise Temperature Monitoring for Firefighting Clothing**

Zhicai Yu^1^, Yuhang Wan^1^, Mi Zhou^1^, Md Hasib Mia^1^, Siqi Huo^2^, Lele Huang^1^, Jie Xu^1^, Qing Jiang^1^, Zhenrong Zheng^3^, Xiaodong Hu^4^, Hualing He^1,^ *****

^1^ State Key Laboratory of New Textile Materials and Advanced Processing, School of Textile Science and Engineering, Wuhan Textile University, Wuhan 430200, P. R. China

^2^ School of Engineering, Centre for Future Materials, University of Southern Queensland, Springfield Central 4300, Australia

^3^ School of Textile Science and Engineering, Tiangong University, Tianjin 300387, P. R. China

^4^ College of Material and Chemical Engineering, Hunan Institute of Engineering, Xiangtan 411104, P. R. China

*Corresponding author. E-mail: [hehualinghe@126.com](mailto:hehualinghe@126.com) (Hualing He)

**ORCID:** orcid.org/0000-0002-0793-7723 (Hualing He)

**S1 Experimental Section**

**S1.1 Preparation of Ag NWs**

Ag NWs were prepared by a typical polyol method. First, 0.01 g of sodium chloride and 0.5 g of PVP were added into 60 mL ethylene glycol, followed by magnetic stirring for 15 min at 700 r/min. Then, 0.51 g of AgNO_3_ was added into the above prepared mixed solution and further stirred for 5 min. Afterward, the prepared mixed solution was transferred into a Teflon-lined stainless autoclave, and then placed in an oven at 180 °C for 8 h and then cooled to room temperature. Finally, the obtained Ag NWs were purified with ethanol centrifuged at 3000 rpm for 15 min, and dried at 60 °C to obtain Ag NWs powders.

**S1.2 Fabrication of MXene nanosheets**

Firstly, the MXene nanosheets dispersion was fabricated through acidic etching of Ti_3_AlC_2_ powers with the mixture of HCl/LiF. In detail, 1.6 g of LiF was slowly added into 20 ml of 12 mol/L HCl followed by magnetic stirring for 40 min to obtain an etching solution in a Teflon container at room temperature. Then, 1 g of Ti_3_AlC_2_ was slowly added into the above prepared etching solution and stirred for 24–36 h to etch the Al layers completely. Afterward, the resultant dispersion was repeatedly washed with distilled water by centrifuging at 3000 rpm for 10 min until the pH of the supernatant was more than 6. The obtained MXene precipitate was further ultrasonicated for 2.5 h to gain the monolayer of MXene nanosheets dispersion. Finally, the as-prepared MXene nanosheets dispersion was centrifuged at 3000 rpm again, followed by freeze-drying to acquire MXene nanosheets.

**S1.3 Functionalization of multi-walled carbon nanotubes (MWCNTs-F)**

Due to the large specific surface area and surface free energy of MWCNTs, it is easy to agglomerate to form large-sized aggregates, which limits the dispersion of MWCNTs in solvents, has poor affinity and weak interfacial bonding. The main methods of dispersing MWCNTs include dispersing carbon nanotubes with surfactants, dispersants or superacids. In this study, MWCNTs were treated with concentrated H_2_SO_4_ and HNO_3_ as oxidants, and carboxyl and hydroxyl functional groups were introduced at the defect site of MWCNTs to enhance their dispersibility. Here, 0.1 g MWCNTs were stirred continuously for 48 h in a mixture of 16 mL H_2_SO_4_/HNO_3_(volume ratio 3:1). Subsequently, the acid-treated MWCNTs were repeatedly filtered at a centrifugal speed of 8000 rpm and washed with deionized water until the filtrate pH reached 7. Finally, the treated MWCNTs were dried at 80 ℃ for 6 h.

**S1.4 Statistical Analysis**

All data are displayed as the means ± standard deviation. In each experiment, at least five replicates were utilized, and data analyses were performed using Origin (2021). The significance difference between the groups was determined via Student’s t-test with the critical *α*-level set at p <0.05.

**S1.5 Measurement of the Density and Porosity of Fibrous Aerogel**

The density of fibrous aerogel mentioned in this manuscript was the “apparent density” rather than the absolute density, which was calculated according to the ISO 845: 2006 standar d: Cellular plastics and rubbers—Determination of apparent density, according to the following formula Eq. S1:

$Apparent density=\frac{Mass of solid constituents}{Volume of test sample}$ (S1)

The free volume (porosity) of fibrous aerogel was determined using the following formula Eq. S2:

$P=\frac{(\rho_{1}-\rho_{0})}{\rho_{1}}\times100$ (S2)

where $P$ is the porosity, $\rho_{0}$(mg·cm^-3^) is the volume density of the samples, and $\rho_{1}$ (mg·cm^-3^) is the density of solid constituents.

**S2 Supplementary Figures and Tables**


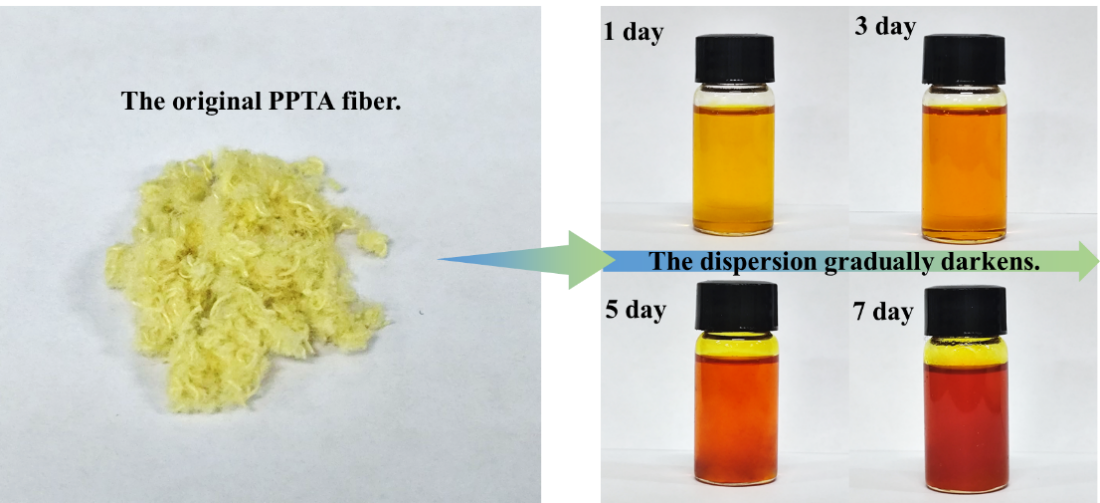


**Fig. S1** Digital photographs of ANFs/DMSO solution at different stirring times


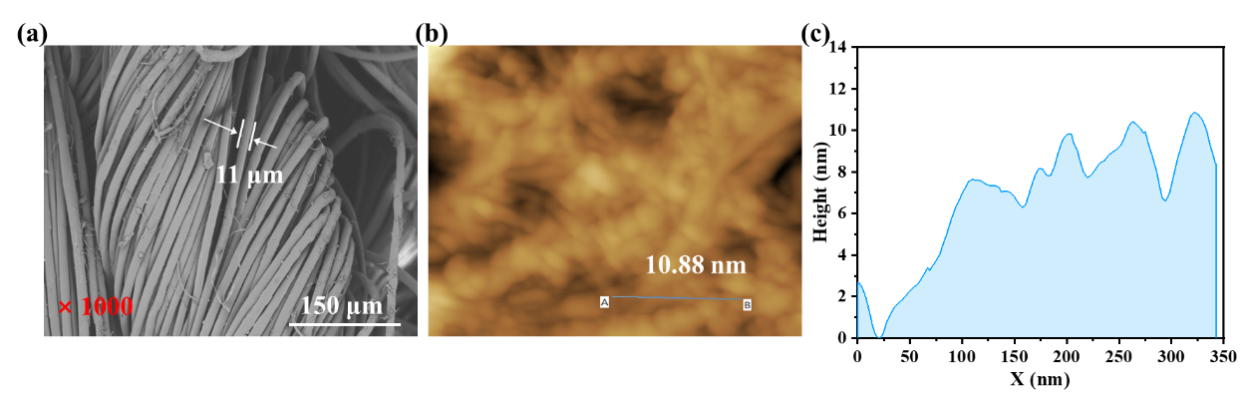


**Fig. S2** **a** SEM showed that the PPTA fibers were between 11 μm in diameter. **b, c** AFM images show that the aramid nanofibers are between 10.88 nm in diameter


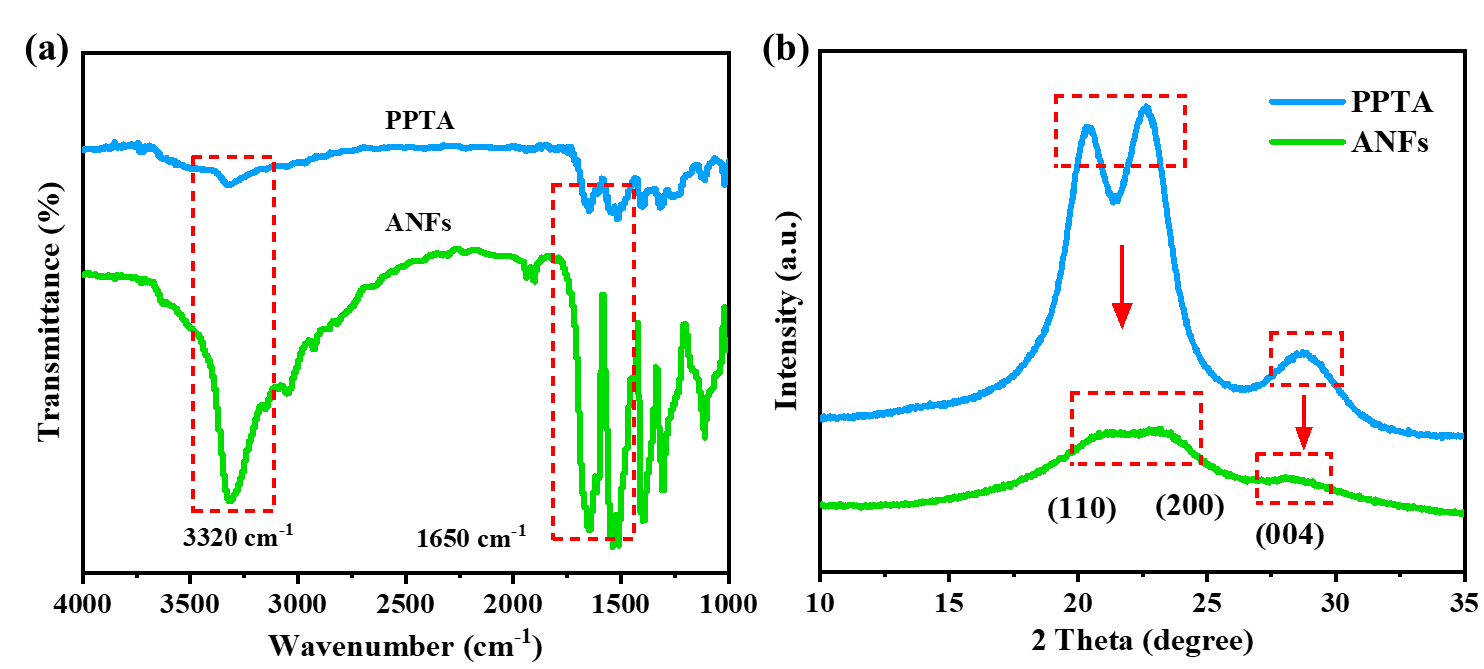


**Fig. S3** **a** FTIR spectra of PPTA fibers before and after dissolution. **b** XRD patterns of PPTA fibers before and after dissolution


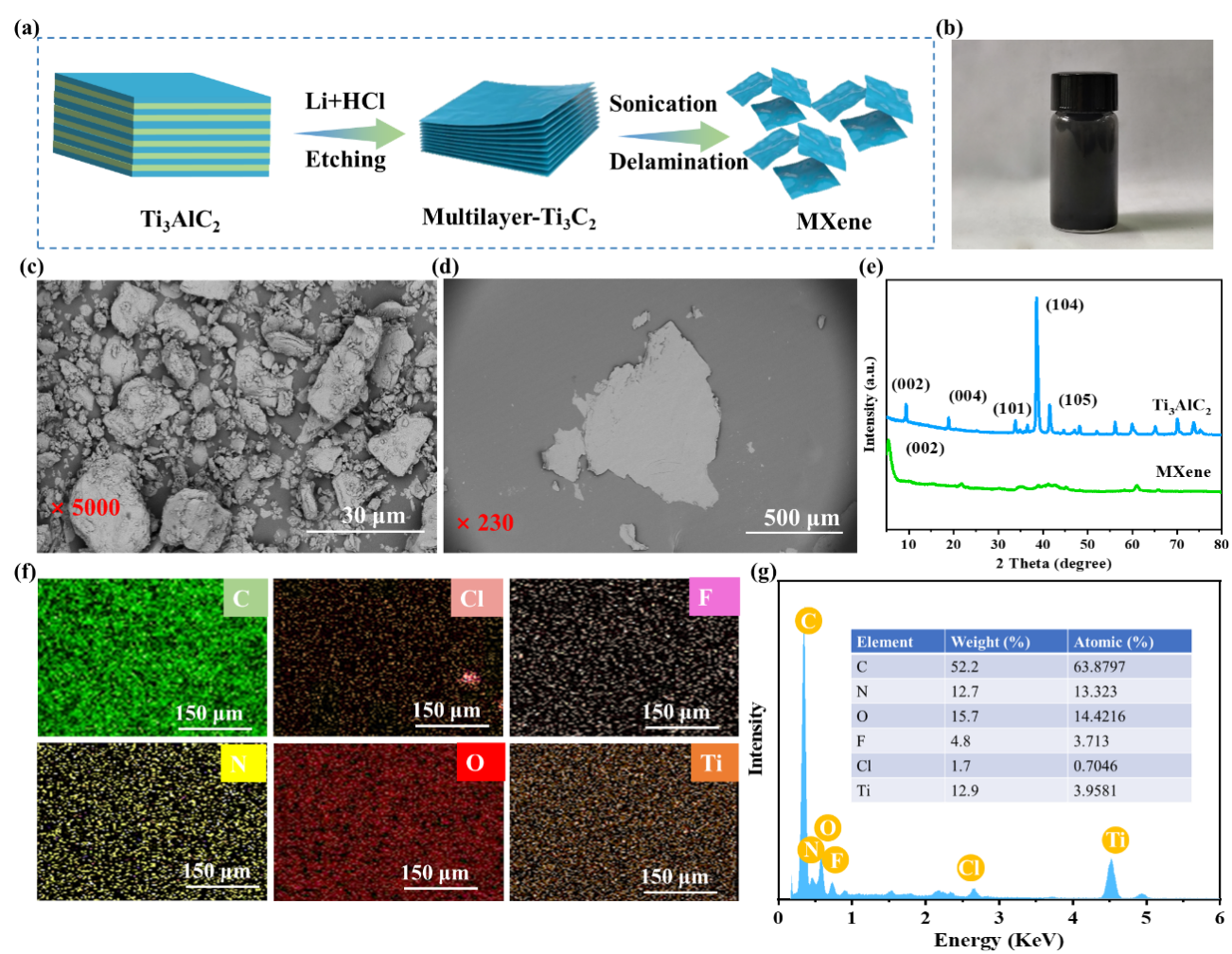


**Fig. S4** **a** Fabrication schematic illustration of MXene nanosheets. **b** The digital image of MXene suspension. SEM images of **c** precursor Ti_3_AlC_2_ powder and **d** the MXene nanosheets with different magnifications. **e** XRD diffraction patterns of Ti_3_AlC_2_ powder and MXene nanosheets. **f** EDX mapping images of different elements on MXene nanosheets surface. **g** EDX spectra image of MXene nanosheets


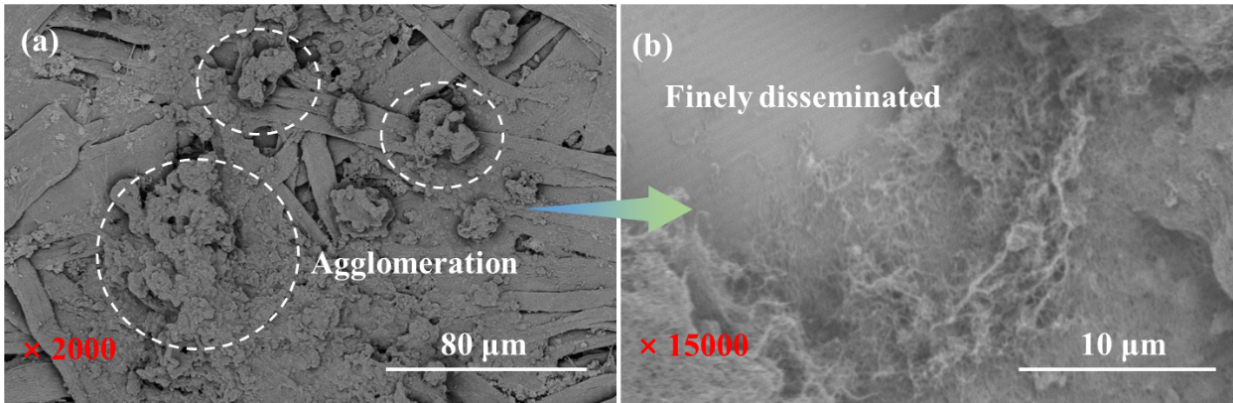


**Fig. S5** SEM images of **a** agglomerated MWCNTs and **b** finely disseminated MWCNTs-F

**Fig. S6** FTIR spectra of MWCNTs and MWCNTs-F


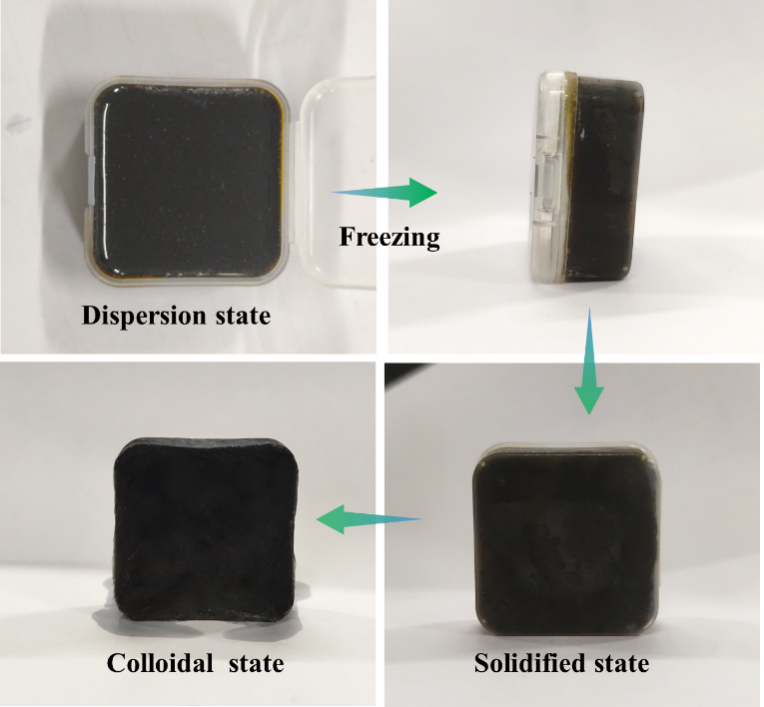


**Fig. S7** ACMCA aerogel undergoes liquid nitrogen directional freezing and changes from liquid state to solid state


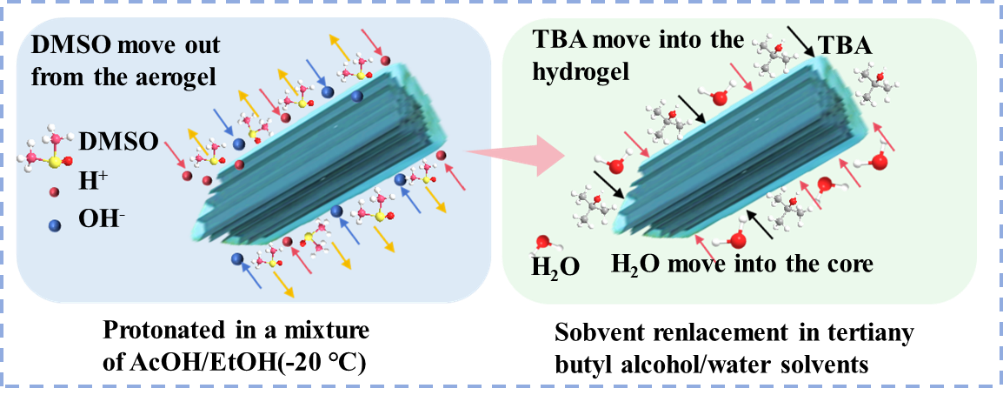


**Fig. S8** Protonation and solvent exchange processes of ACMCA aerogel precursors


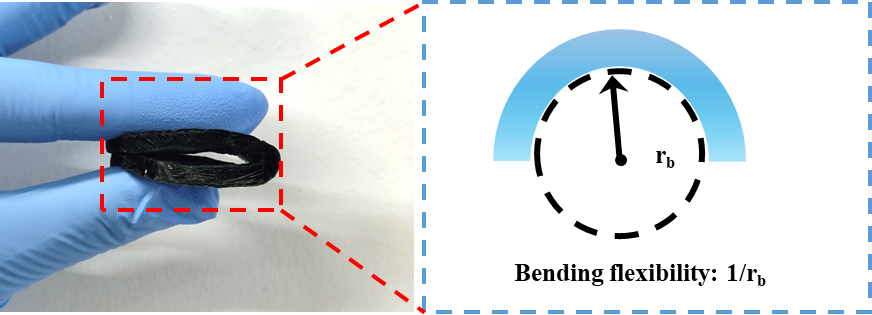


**Fig. S9** ACMCA aerogel with a thickness of 8 mm can withstand large bending deformation, the minimum bending radius (r_b_) of the aerogel is 2 mm, and the bending flexibility is 0.5 mm^-1^


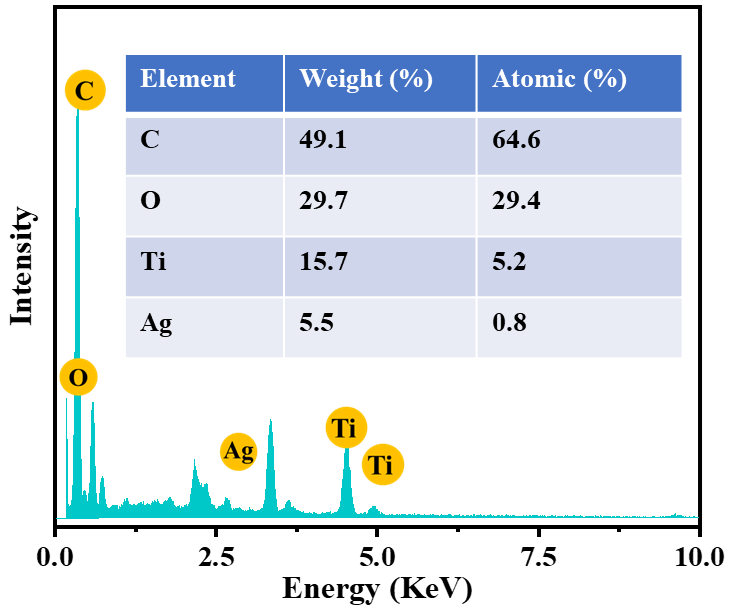


**Fig. S10** EDX spectra image of ACMCA aerogel


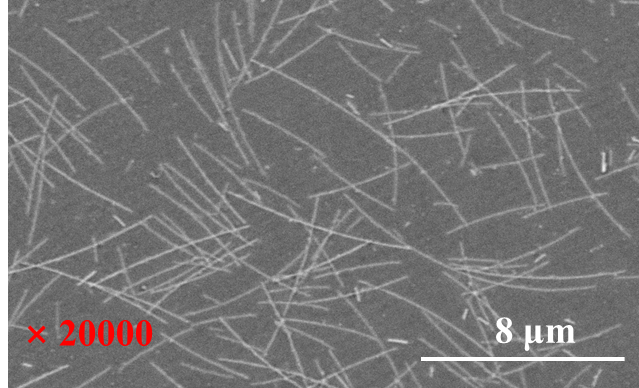


**Fig. S11** SEM image of prepared silver nanowires by polyol method

**Fig. S12** XRD spectra of Eicosane

**Fig. S13** FTIR image of Mxene

**Fig. S14** FTIR spectra of Eicosane


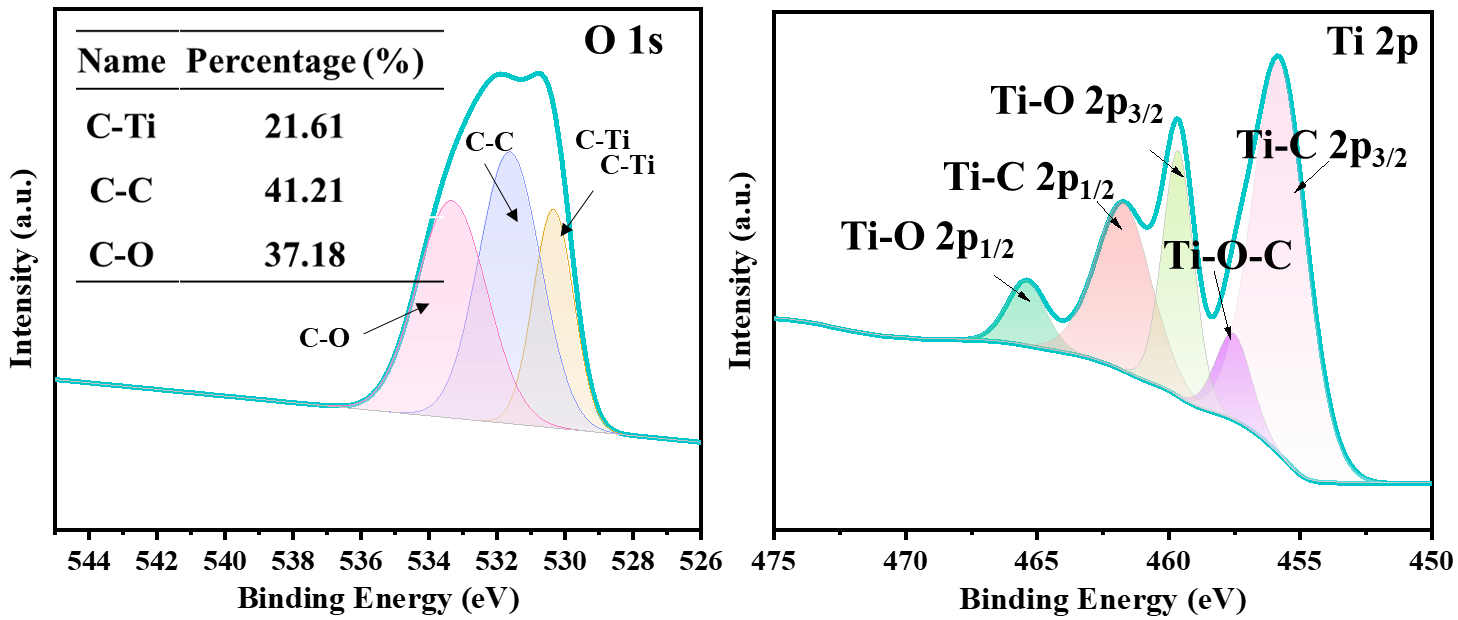


**Fig. S15** High-resolution XPS spectra of O 1s and Ti 2p for MXene/MWCNTs-F sample


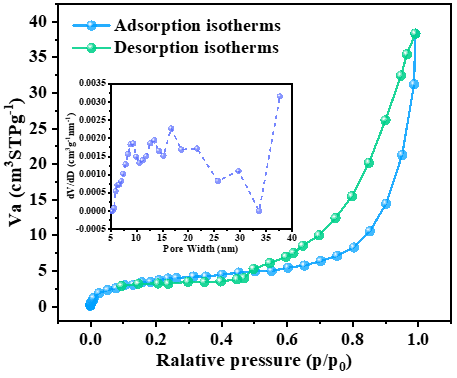


**Fig. S16** N_2_ adsorption-desorption curve and pore size distribution curve of ACMCA aerogel


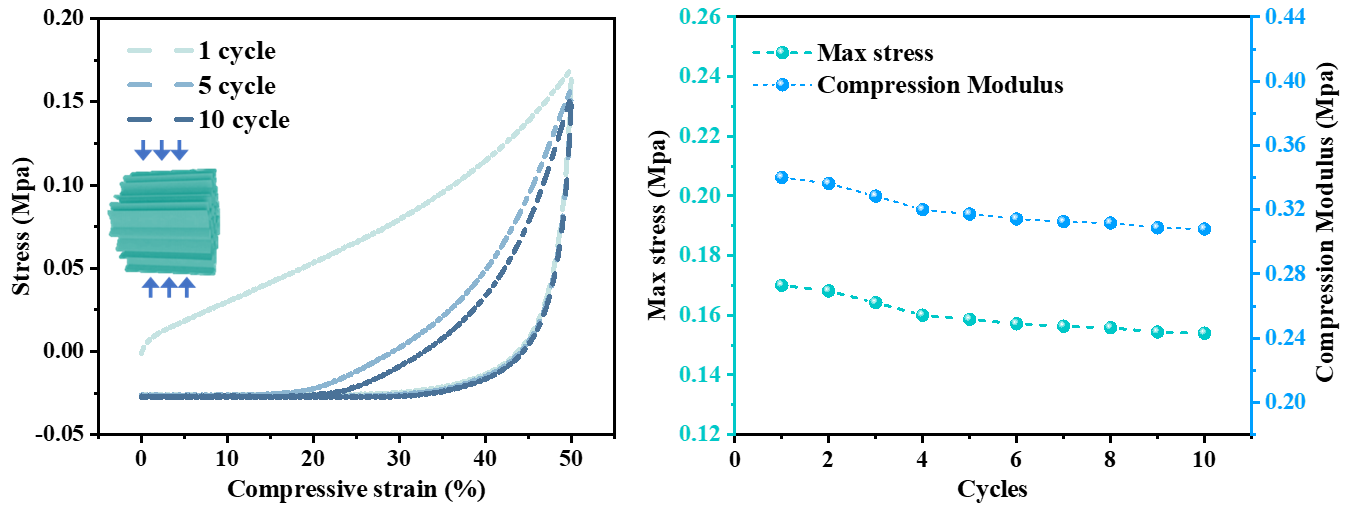


**Fig. S17** Radial cyclic compression curve of ACMCA aerogel and corresponding maximum stress and compression modulus

**
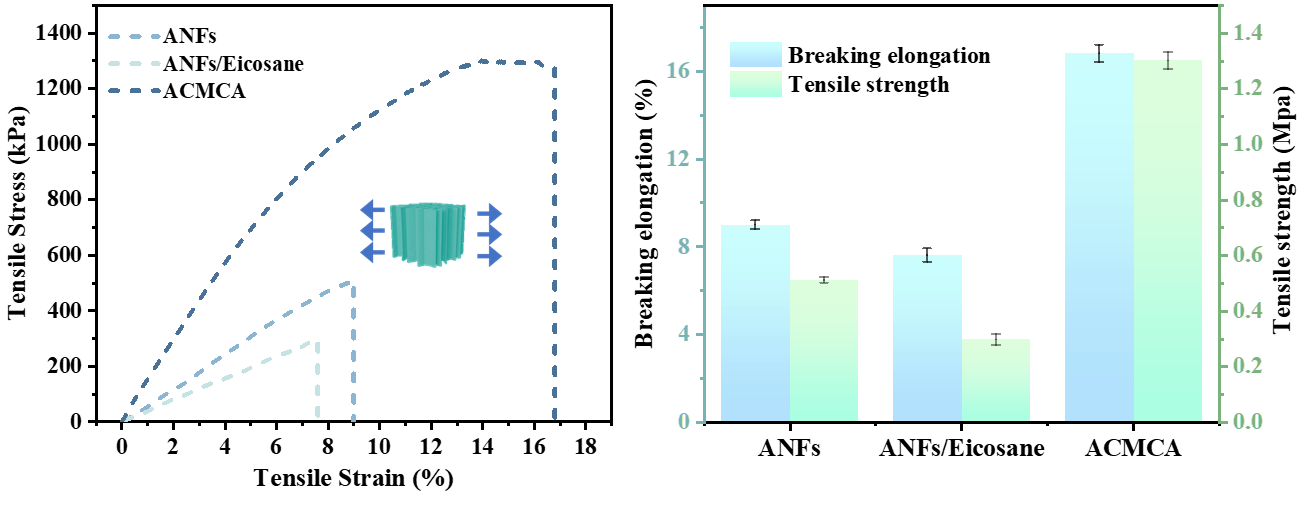
**

**Fig. S18** ANF, ANF/Eicosane and ACMCA aerogels radial tensile curve and corresponding elongation at break and tensile strength


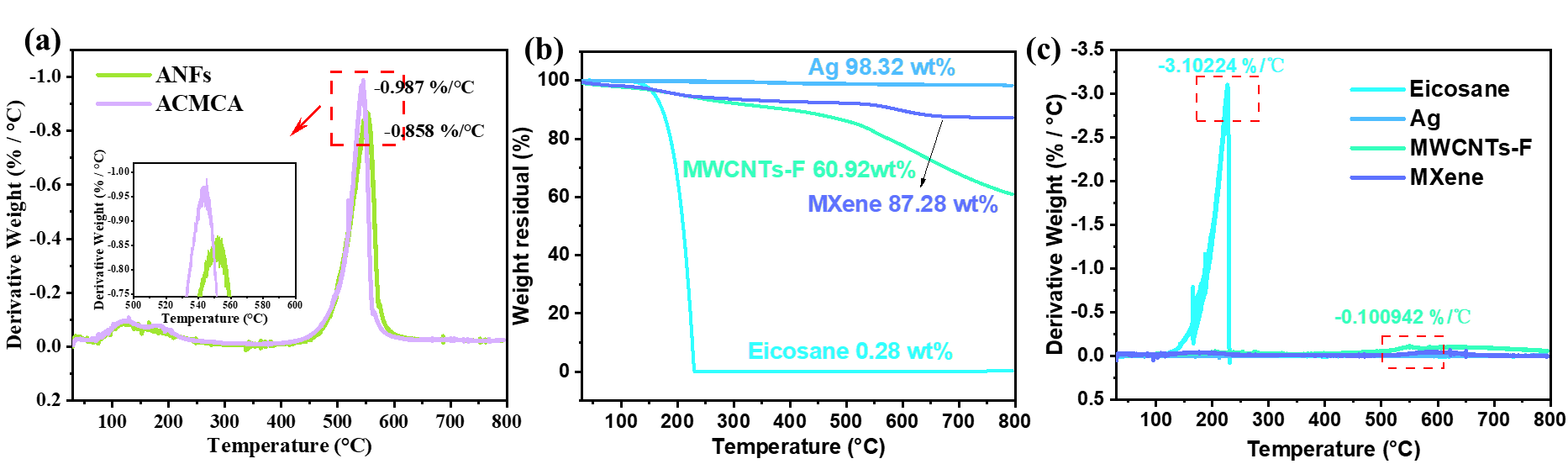


**Fig. S19 a** DTG curves of ANFs and ACMCA in a nitrogen atmosphere. **b** TG and **c** DTG curves of Ag, MWCNTs-F, MXene, and Eicosane in a nitrogen atmosphere


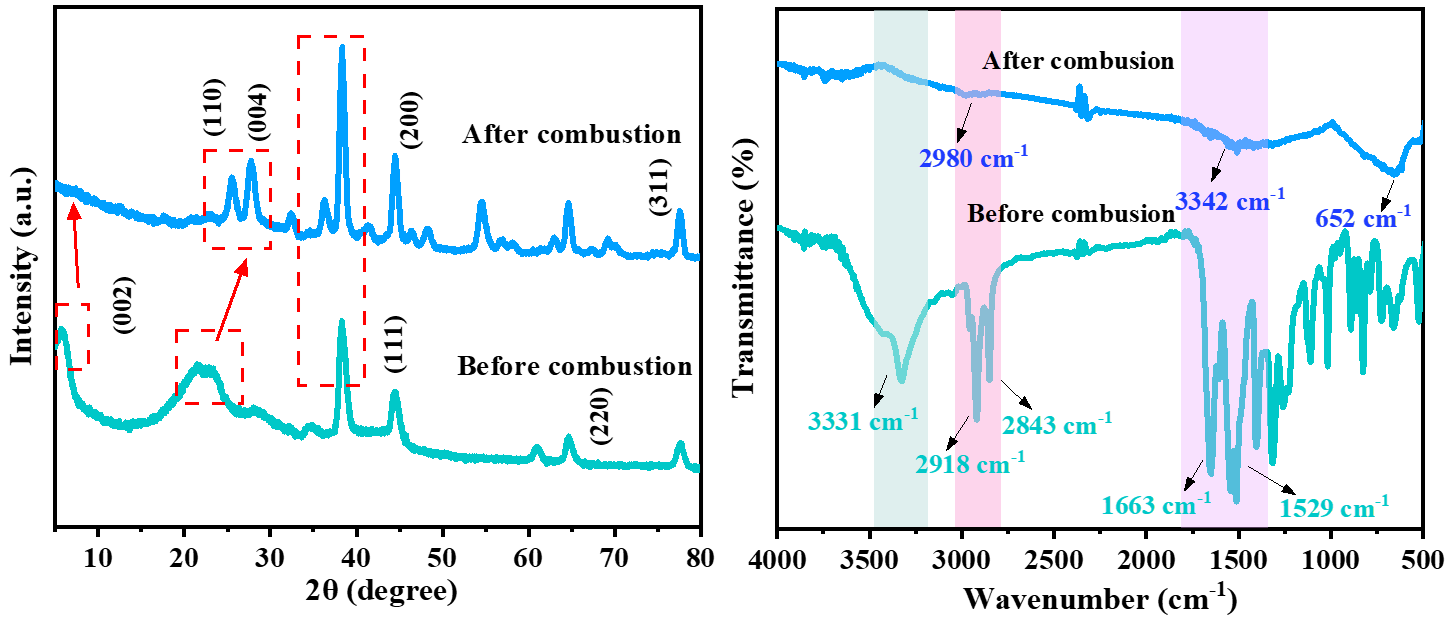


**Fig. S20** XRD and FTIR images of ACMCA aerogel before and after combustion

**Fig. S21** 3D TG-FTIR spectra of the ACMCA aerogel


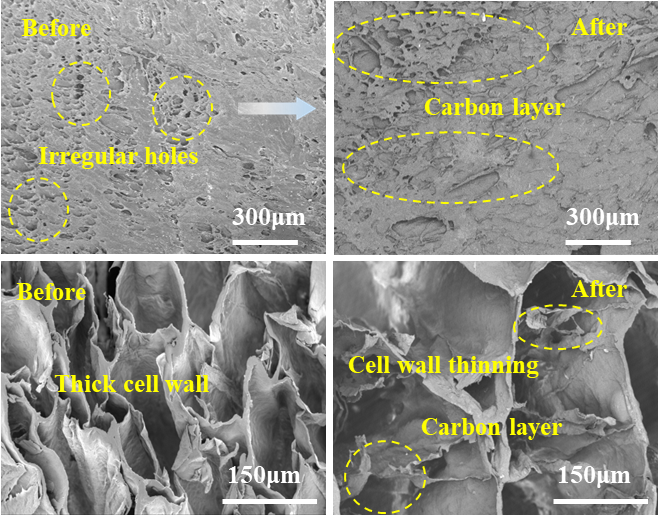


**Fig. S22** SEM images of surface and cross sections of ACMCA aerogel before and after combustion in an alcohol lamp


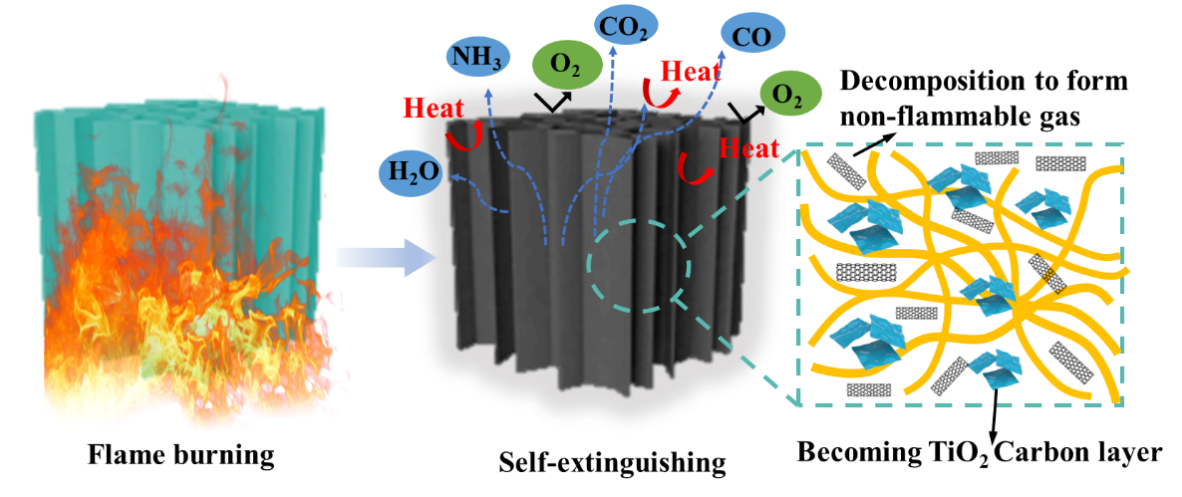


**Fig. S23** Schematic diagram of flame retardant mechanism of ACMCA aerogel


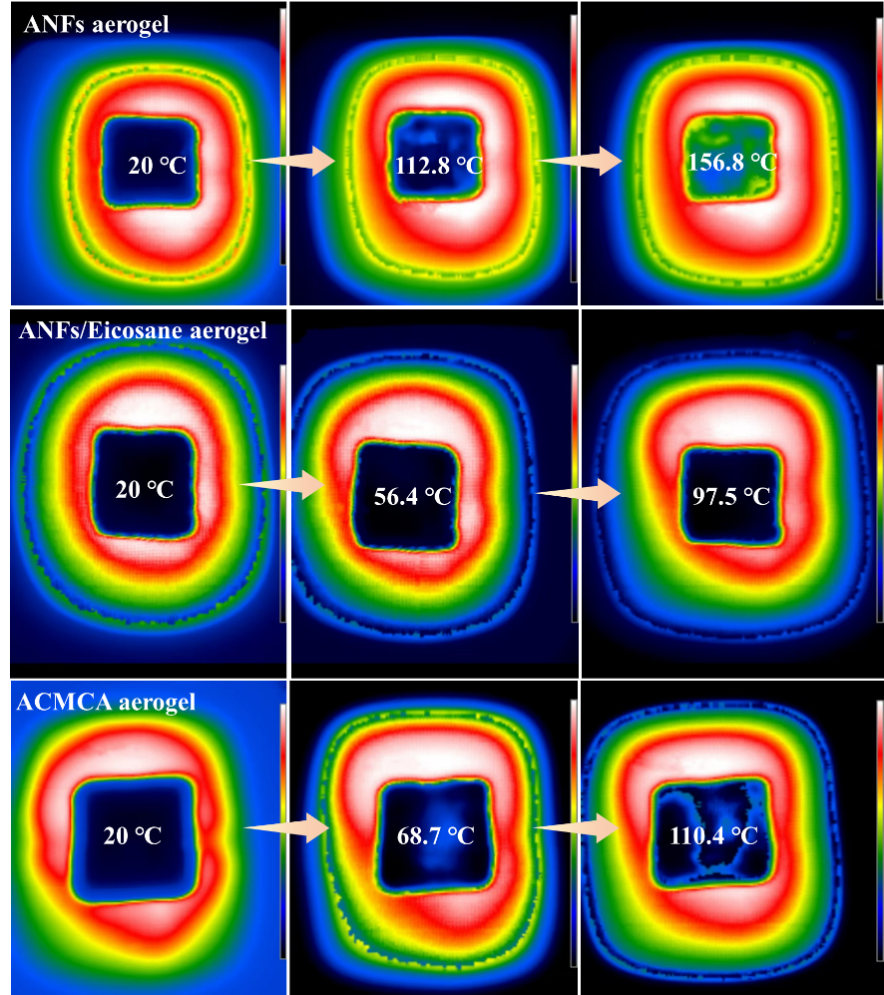


**Fig. S24** Infrared thermal image of ANFs, ANFs/Eicosane and ACMCA aerogel on a 300 ℃ heating plate


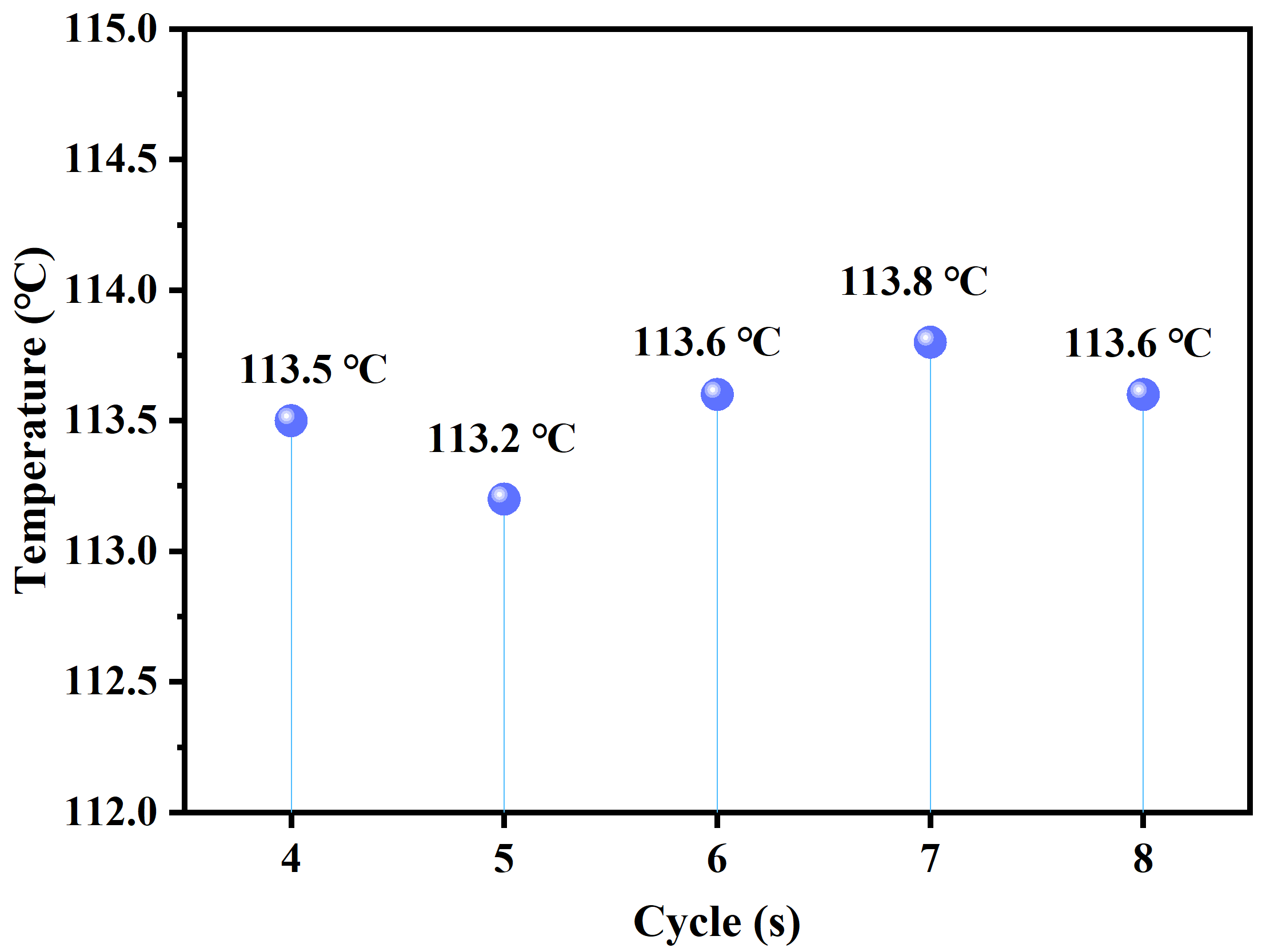


**Fig. S25** Balance temperature of the upper surface of ACMCA aerogel after cyclic heating and cooling on the heating plate.


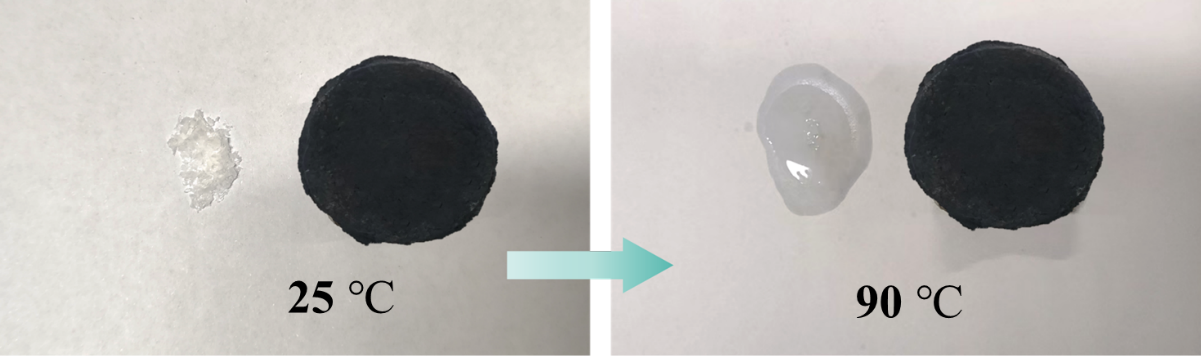


**Fig. S26** Eicosane leakage experiment was carried out for Eicosane and ACMCA aerogel

**
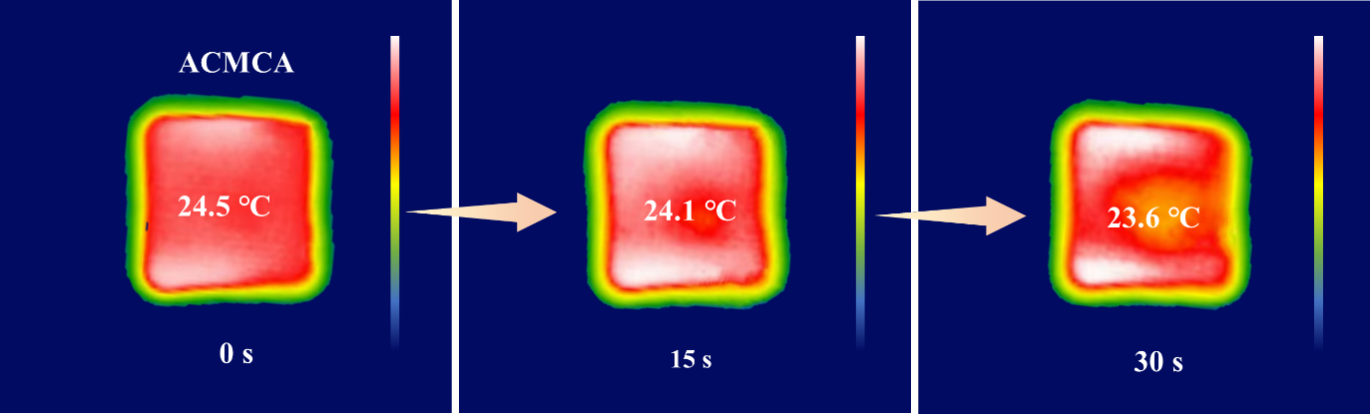
**

**Fig. S27** Thermal infrared images of ACMCA aerogel placed in a cold environment for 30 s

**Fig. S28** Histogram of the density and thermal conductivity of ANFs, ANFs/Eicosane and ACMCA aerogels


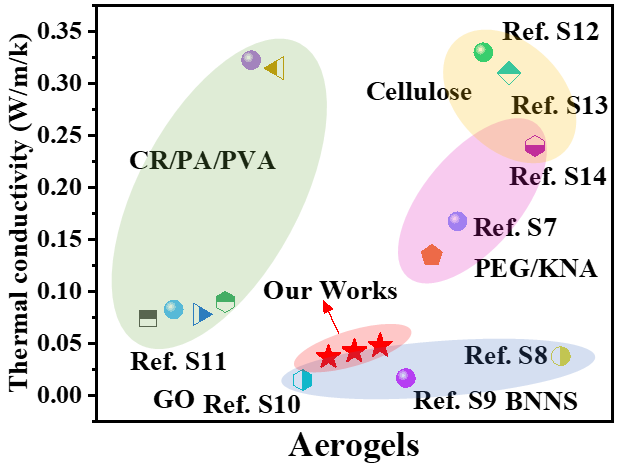


**Fig. S29** Comparison of thermal conductivity in this work with reported composite aerogels [S1-S8]


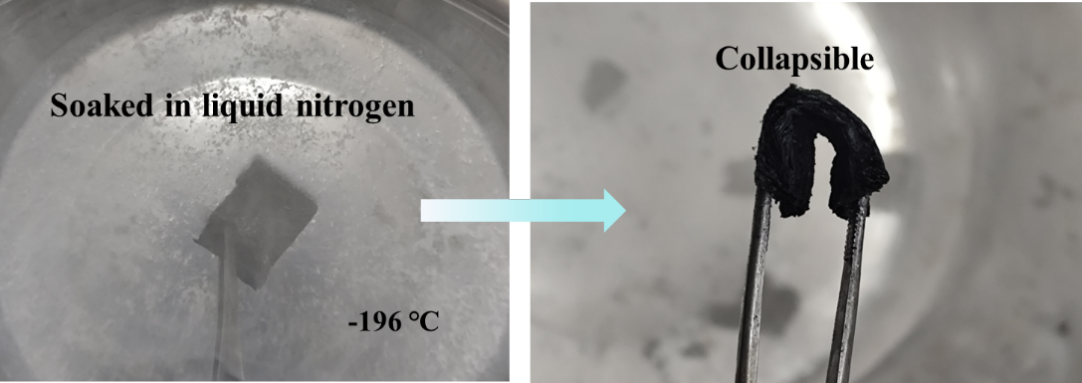


**Fig. S30** Flexibility and immutability in extremely cold environments

**Fig. S31** Compared with traditional porous materials, ACMCA aerogel have a smaller pore size

**Fig. S32** The output voltage vs time curves of the prepared aerogels by directional freezing ACMCA and non-directional freezing Non-ACMCA on a 400 ℃ heating plate

**Fig. S33** The fitted curve of the output voltage of the Non-ACMCA aerogel at different temperatures


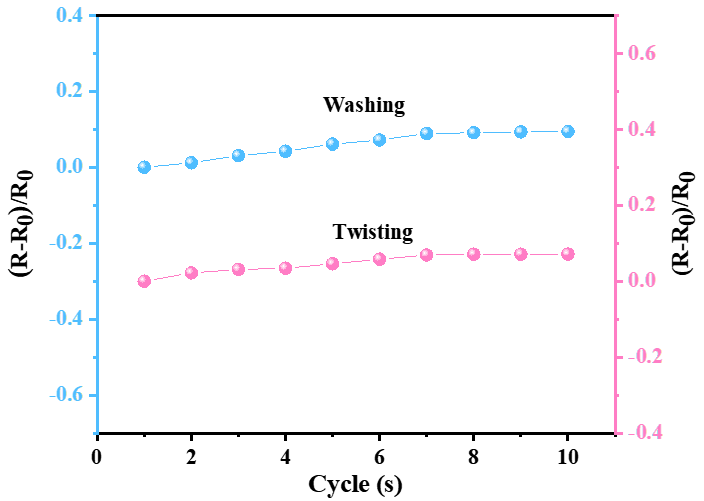


**Fig. S34** The durability test of ACMCA aerogel under different washing times and twisting times at room temperature


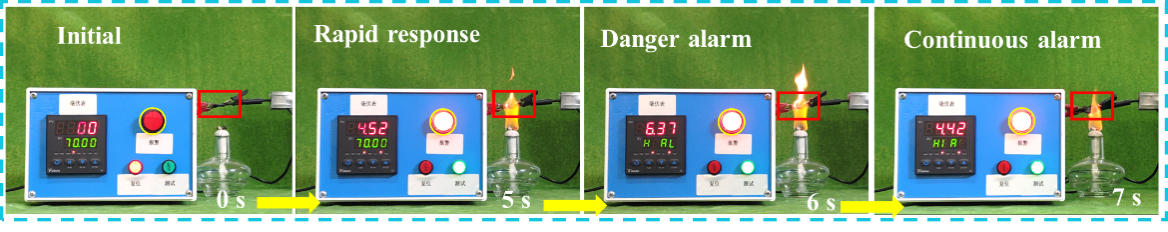


**Fig. S35** Self-powered Non-ACMCA triggers a warning for 5.2 s when exposed to flames


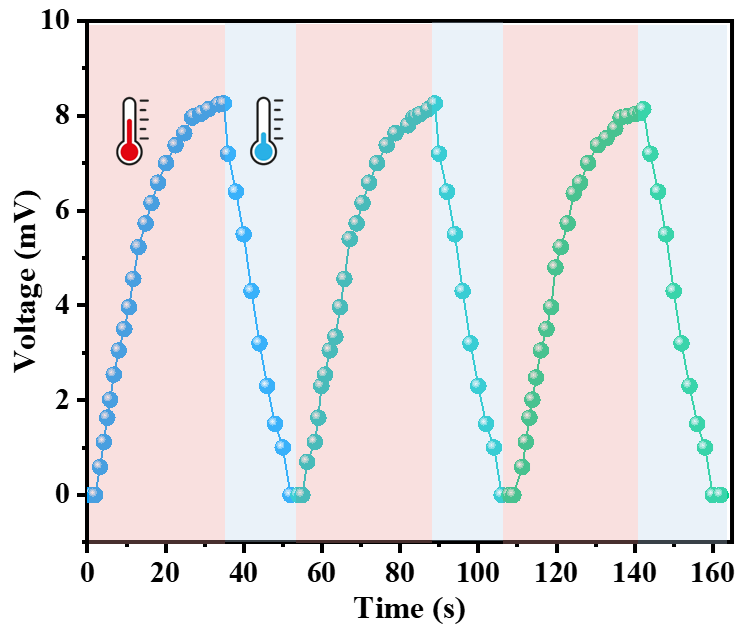


**Fig. S36** Cyclic output voltage performance of ACMCA when intermittent exposed to

flame of the alcohol lamp


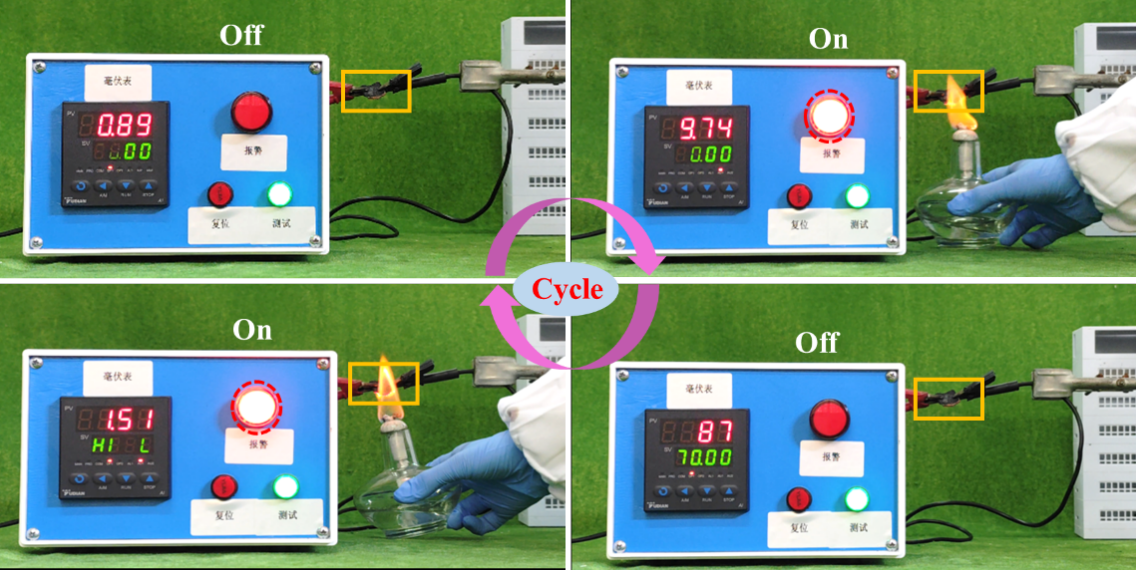


**Fig. S37** Reversible fire warning capability of ACMCA when intermittently exposed to the flame


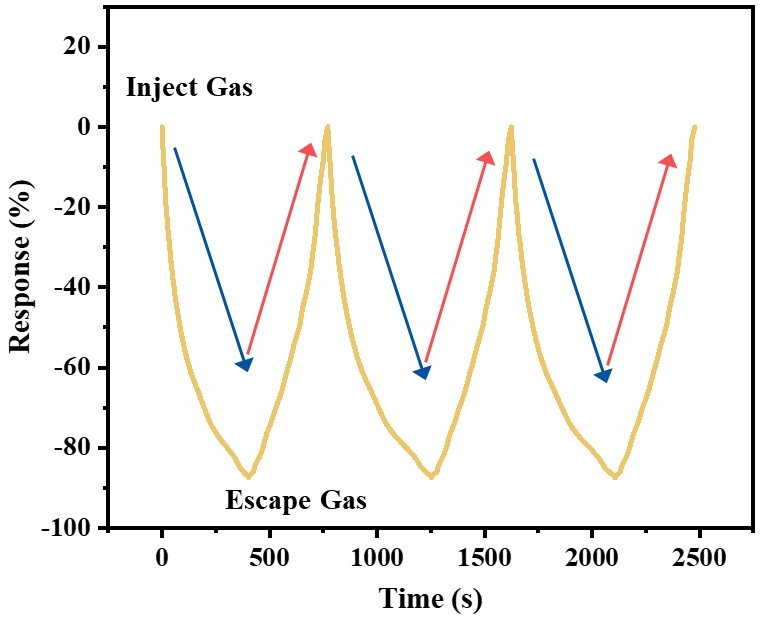


**Fig. S38** Reversible ammonia detection function of ACMCA-M aerogel


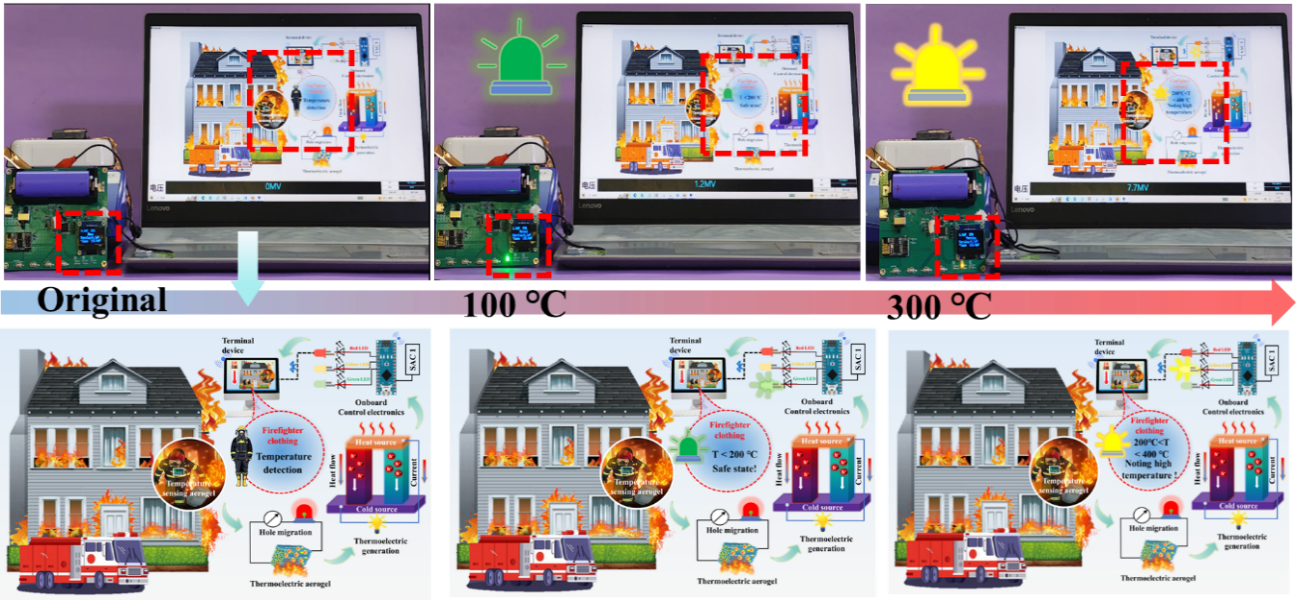


**Fig. S39** ACMCA-based self-driven multistage wirelessalarm alarm system for high temperature warning of firefighting clothing via different colors of LED lights indicating “Safe” state, “Noting high temperature” state, and “Warning” state

**Fig. S40** The resistance change rate of ACMCA under cyclic compression with a compression strain of 20%


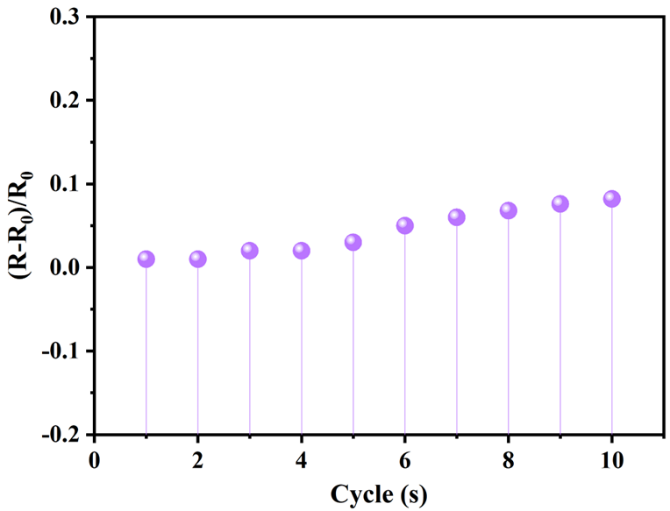


**Fig. S41** Changes in electrical resistivity after compression cycles at 10% compression strain under different compression cycles


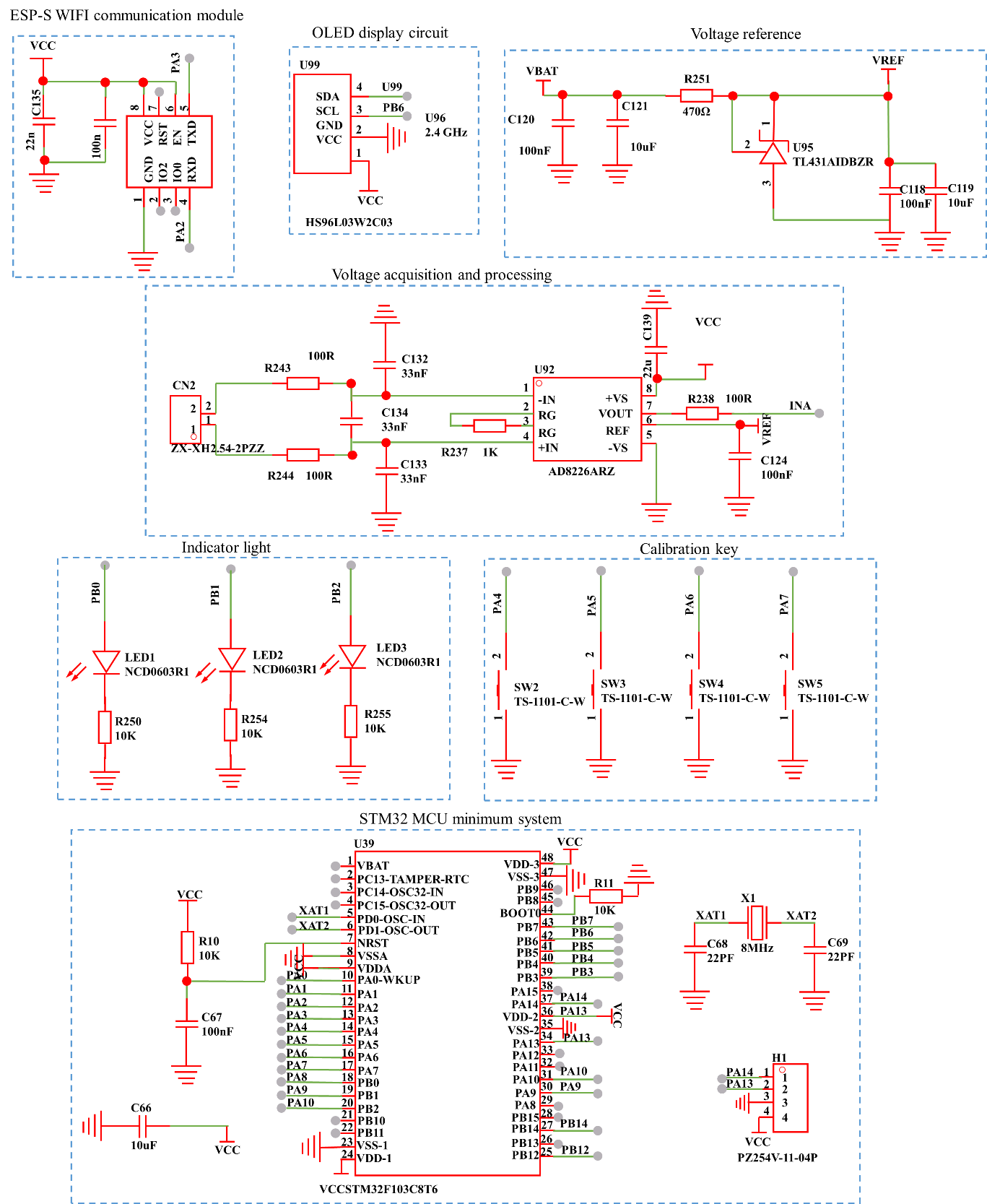


**Fig. S42** Electronic design automation schematic diagram of different modules of wireless early high temperature warning system


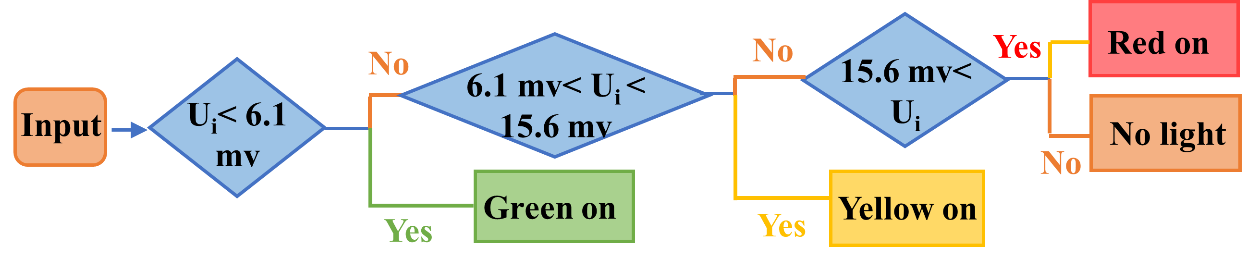


**Fig. S43** Logical algorithm was performed to demonstrate high temperature warning function of ACMCA aerogel

**Table S1** Characteristic parameters of TGA and DTG for aerogels under nitrogen atmosphere

| Atmosphere | Sample | T_i_  (°C) | T_max_^-1^  (°C) | R_max_^-1^  (wt%·min^-1^) | Residue at 800 °C (wt%) |
| --- | --- | --- | --- | --- | --- |
| Nitrogen | ANFs | 109.61 | 551.135 | 0.86444 | 33.57 |
|  | ACMCA | 118.979 | 545.108 | 0.987 | 36.92 |
|  | Eicosane | 159.64 | 227.017 | 3.10224 | 0.28 |
|  | Ag | / | 29.68 | 0.035087 | 98.32 |
|  | MWCNTs-F | 208.69 | 556.105 | 0.100942 | 60.92 |

Note: T_i_, T_max_^-1^ and R_max_^-1^ are respectively the temperature at 95% weight loss, the temperature at maximum weight loss rate and the maximum weight loss rate of the aerogel during the TG test.

**Table S2** Thermophysical parameters of PEG and ACMCA derived from DSC analysis

| Sample | Melting process | |  | Crystallization process | |
| --- | --- | --- | --- | --- | --- |
|  | T_pm_(℃) | ΔH_m_ (J·g^-1^) |  | T_pc_(℃) | ΔH_c_ (J·g^-1^) |
| Eicosane | 40.8 | 175.8 |  | 27.1 | 182.7 |
| ACMCA | 45.7 | 156.3 |  | 28.9 | 156.9 |

Note: T_om_, T_pm_ and T_em_ are the starting temperature, peak temperature and end temperature of aerogels melting, respectively. T_oc_, T_pc_ and T_ec_ are the starting temperature, peak temperature and end temperature of aerogel crystallization, respectively.

**Supplementary Movie S1:** Flame retardant property testing of ACMCA aerogel with alcohol lamp. **Description:** The combustion behavior of ACMCA aerogel was further investigated using alcohol lamps. The ACMCA aerogel maintained original shape without any change after 12 s of combustion, and the ACMCA aerogel showed significant self-extinguishing performance after the alcohol lamp was removed.

**Supplementary Movie S2:** Reversible high temperature warning test of ACMCA aerogel (Cycle 4).

**Description:** The movie showed that the ACMCA aerogel still triggered the fire alarm light after alternate contact with the flame four times, indicating reversible high temperature warning capability.

**Supplementary Movie S3:** Multistage wireless high temperature warning test.

**Description:** In the temperature monitoring and early warning system, we established predetermined thresholds for high and low temperatures, using three different colors of LED lights as indicators for safety status, attention status, and danger alarm status. To implement the temperature alarm function, the ACMCA aerogel was fixed on the temperature control platform, and the temperature conversion process monitored by the early warning system was simulated through the heating and cooling mechanisms of the control platform. In the absence of temperature, everything remained calm. However, as soon as the temperature was detected, the early warning system was activated and showed different levels of warning. When the aerogel was below 200 ℃, the green light prompt was triggered, indicating a “safe state,” Between 200 °C and 400 °C, the “Noting high temperature” was triggered. When the temperature exceeded 400 °C, the red light was triggered, and the “Warning state” was displayed.

**Supplementary References**

1. Y. Meng, J. Majoinen, B. Zhao, O. Rojas, Form-stable phase change materials from mesoporous balsa after selective removal of lignin. Compos. Pt. B-Eng. **199**, 108296 (2020). <https://doi.org/10.1016/j.compositesb.2020.108296>
2. J. Zhu, S. Jiang, H. Hou, S. Agarwal, A. Greiner, Low density, thermally stable, and intrinsic flame retardant Poly(bis(benzimidazo)Benzophenanthrolinedione) sponge. Macromol. Mater. Eng. **303**, 1700615 (2018). <https://doi.org/10.1002/mame.201700615>
3. K.-Y. Chan, X. Shen, J. Yang, K.-T. Lin, H. Venkatesan et al., Scalable anisotropic cooling aerogels by additive freeze-casting. Nat. Commun. **13**, 5553 (2022). <https://doi.org/10.1038/s41467-022-33234-8>
4. B. Wicklein, A. Kocjan, G. Salazar-Alvarez, F. Carosio, G. Camino et al., Thermally insulating and ﬁre-retardant lightweight anisotropic foams based on nanocellulose and graphene oxide. Nat. Nanotechnol*.* **10**, 277 (2015). <https://doi.org/10.1038/nnano.2014.248>
5. W. Xie, J. Wang, K. Shang, H. Chen, H. Chen et al., A nanoclay-based 3D aerogel framework for flexible flame retardants. Mater. Adv. **3**, 6799 (2022). <https://doi.org/10.1039/d2ma00556e>
6. Y. Liu, Y. Zhang, T. Liao, L. Gao, M. Wang et al., Boron nitride–nanosheet enhanced cellulose nanoﬁber aerogel with excellent thermal management properties. Carbohydr. Polym*.* **241**, 116425 (2020). <https://doi.org/10.1016/j.carbpol.2020.116425>
7. J. Shi, L. Lu, W. Guo, J. Zhang, Y. Cao, Heat insulation performance, mechanics and hydrophobic modiﬁcation of cellulose–SiO_2_ composite aerogels. Carbohydr. Polym. **98**, 282 (2013). <http://dx.doi.org/10.1016/j.carbpol.2013.05.082>
8. F. He, Z. Qi, W. Zhen, J. Wu, Y. Huang et al., Thermal conductivity of silica aerogel thermal insulation coatings. Int. J. Thermophys. **40**, 92 (2019). <https://doi.org/10.1007/s10765-019-2565-6>
